# Facile and Sustainable Synthesis of Shaped Iron Oxide Nanoparticles: Effect of Iron Precursor Salts on the Shapes of Iron Oxides

**DOI:** 10.1038/srep09733

**Published:** 2015-05-05

**Authors:** Farheen N. Sayed, Vivek Polshettiwar

**Affiliations:** 1 Nanocatalysis Laboratory (NanoCat), Department of Chemical Sciences, Tata Institute of Fundamental Research (TIFR), Mumbai, India

## Abstract

A facile and sustainable protocol for synthesis of six different shaped iron oxides is developed. Notably, all the six shapes of iron oxides can be synthesised using exactly same synthetic protocol, by simply changing the precursor iron salts. Several of the synthesised shapes are not reported before. This novel protocol is relatively easy to implement and could contribute to overcome the challenge of obtaining various shaped iron oxides in economical and sustainable manner.

Not only the size, but shape of nanomaterials exerts tremendous impact on their properties, including catalysis^1^,^2^. Shape changes the exposed crystal facets and hence the atomic arrangements in each facets which will have profound effect on its various properties. Also, for the development of economical and sustainable nano-catalysts, use of non-precious metal based nanomaterials such as iron oxides as a catalyst is the key^1^,^2^. The magnetic property of iron oxide; being an additional feature makes it potential candidate in applications where effective separation of the material is must. Moreover, activation of iron oxide by changing its particle shape to expose its most active catalytic site could yield cheap and efficient catalysts for various reactions. Along with the change in shape, the phase dependent properties can also be fine-tuned by varying synthesis parameters. First step in achieving these objectives is to develop facile and economic synthetic protocol for iron oxides with range of shapes.

Many successful attempts has been made to synthesis shaped iron oxides using different mechanochemical (electrodeposition, laser ablation arc discharge, pyrolysis, combustion) and chemical (sol-gel synthesis, reverse micelle, co-precipitation, template assisted synthesis and hydrothermal) methods^3^,^4^,^5^,^6^. By employing solvothermal techniques, various morphologies of iron oxides like nanorods, rings, cubes, spindles, hollow particles etc. have been prepared^7^,^8^,^9^,^10^,^11^,^12^.

Despite the intense effort that has been currently ongoing, it is still a big challenge to develop facile and sustainable synthetic protocol for the controlled synthesis of shaped iron oxides. In continuation of our work on synthesis of shape and morphology controlled nano-material synthesis^13^,^14^,^15^, here, we report facile and sustainable synthesis of iron oxides with six different shapes (Fig. 1), using our modified KCC-1 synthesis protocol^16^,^17^,^18^,^19^,^20^,^21^, a microwave (MW) assisted templated solvothermal technique. Significantly, all the six shapes of iron oxide can be synthesised using exactly same synthetic protocol, by simply changing the iron salt. To the best of our knowledge, developed protocol as well as several of these shaped iron oxides are unique and not reported before in the literature. The prepared samples were thoroughly characterized for their textural properties such as; size, shape, phases, surface area, etc. Bandgap measurements along with the magnetic properties evaluation were also studied.

## Results and Discussion

The six shapes of iron oxide was synthesised using KCC-1 synthesis protocol^16^,^17^,^18^,^19^,^20^,^21^, which involves various iron salts as iron-precursors, cetyltrimethlammonium bromide (CTAB) as a template, cyclohexane-water-pentanol as a reaction solvent and urea as hydrolysing agent. MW assisted heating of reaction mixture at various temperature yielded various shaped iron oxides.

### Nanorods of Iron Oxides

**Figure 2:**
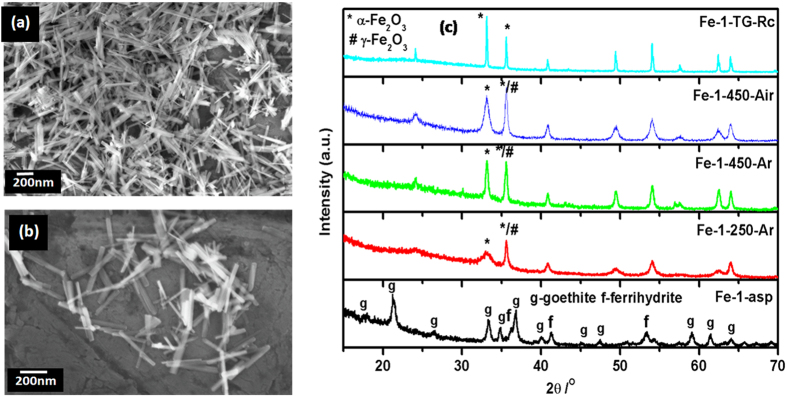
(**a**-**b**) SEM images of as-prepared nanorods (Fe-1-asp) (**c**) XRD pattern of as-prepared nanorods along with the different heat treated nanorods.

With Fe(II) sulphate as the precursor, rod shaped nanoparticles have been observed (Fig. 2a-b). The average length of the nanorod was in the range of 270–315 nm with the width of 30–35 nm range. XRD pattern (Fig. 2c) suggests that the as-prepared sample consists of mainly α-FeOOH (goethite), which matches very well with the standard XRD pattern (PCPDF 29-0713).

**Figure 3:**
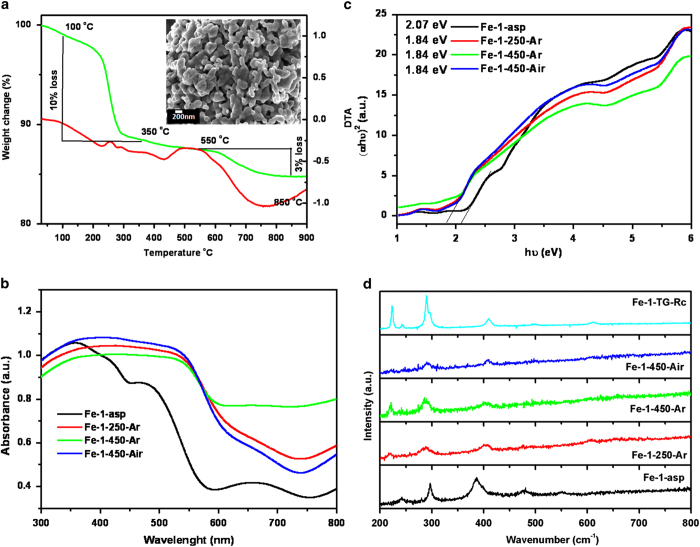
(**a**) TG/DTA curves of Fe-1-asp sample, inset shows the SEM image of Fe-1-TG-Rc sample; (**b**) Absorption spectra (**c**) Tauc Mott plots and (**d**) Raman spectra of as-prepared as well as heat treated samples.

The formation of this goethite phase is facilitated by the olation or condensation of hydroxo and aquo-hydroxo complexes in solution^22^. Along with the goethite phase, peaks at 37^o^, 42^o^ and 54^o^ corresponding to 5Fe_2_O_3_.9H_2_O (PCPDF 29-0712), were also observed. The thermal stability and decomposition behaviour of as-prepared sample was studied by thermo-gravimetric and differential thermal analysis (TG/DTA) in nitrogen atmosphere. The total observed weight loss of 16% comprising of two steps is seen in Fig. 2a. The weight loss in the temperature range of 100 to 400 ^o^C corresponds to the decomposition of the organic surfactant (CTAB) as well as removal of the lattice water from ferrihydrite. This was also reflected in DTA curve as a small endothermic peak. The further weight loss in the range of 550 to 850 ^o^C indicated some structural rearrangement or transition occurring in the system. The residue obtained after this thermal treatment Fe-1-TG-Rc found to be fully transformed into pure α-Fe_2_O_3_ phase, as seen in XRD pattern (Fig. 2c) To further investigate the temperature dependent phase transitions, the as-prepared sample was heated at different temperature in ambient as well as in inert atmosphere. The XRD patterns of the heated samples are given in Fig. 2c. It was observed that at lower temperature, sample consists of mixture of α and γ-Fe_2_O_3_, which on heating gradually transforms to pure α-Fe_2_O_3_ (PCPDF 33-0664). It is clearly visible that as heating temperature increases, the peaks corresponding to the α-Fe_2_O_3_ increases, leading to complete conversion to pure α-Fe_2_O_3_ at high temperature. While comparing the air heated and argon heated samples at 450 ^o^C, (Fe-1-450-Air and Fe-1-450-Ar), it is evident that there is significant change in ratio of the peaks corresponding to the α-Fe_2_O_3_ and γ-Fe_2_O_3_. The effect of these heat treatments on the morphology was also investigated by SEM analysis. The SEM images reflect that effect of heating in air is not as drastic as on heating in inert atmosphere (SI-1). 250 ^o^C heated sample (Fe-1-250-Ar) shows increase in the width of nanorods but no change in length, although this effect was not uniform. In case of 450 ^o^C heated sample (Fe-1-450-Ar), there was stark effect on the particle shape. The nanorods diffuses into each other forming a glassy phase, which is more stable shape than compared to nanorods, in terms low free energy and surface charge. Similarly, the SEM image of the Fe-1-TG-Rc showed the final stage of the diffusion (Fig. 3a (inset)). The observed morphology and phases for the as-prepared as well as heated samples are compared in Table 1.

The as-prepared and heat treated samples were also characterized for their electronic structure by diffuse reflectance UV-Vis studies (Fig. 3b). The diffuse reflectance (DR) UV-Vis spectrum of iron based hydroxides/oxides can be divided into four regions based on absorptions; ligand to metal charge transfer (250–400 nm) along with contribution of Fe^3+^ ligand field transition (290–310 nm), pair excitation process (400–600 nm) of magnetically coupled Fe^3+^ ions and two strong absorption bands near 640 and 900 nm of ligand field transitions of Fe^3+^ cation in octahedral environment^23^. Fe-1-asp, which is the mixture of α-FeOOH and ferrihydrtie (as observed in XRD pattern), showed the well resolved low energy peaks at 655 and 950 nm but high energy transition peaks were poorly resolved. The peaks at 289 and 368 nm corresponding to the 6A_1_-4T_1_(4P) transition of pure goethite structure overlaps with the band at 250 nm of ferrihydrite assigned to 6T_1u_-6T_1u_. Along with the as-prepared sample, the heat treated samples also showed similar features in the absorption spectra. The broad absorption with the edge at 535 nm was observed, which is associated with the double excitation process 6A_1_(6S)-6A_1_(6S) to 4T_1_(4G)-4T_1_(G). This double excitation process is also responsible for the red color of α-Fe_2_O_3_ phase.

Tauc Mott plot is used to calculate the bandgap of these materials. The Tauc formula depicting the relationship between absorption coefficient and incident photon is as follows:


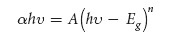


Where, α is absorption coefficient, A is constant and n can be 2 (direct transition) or 0.5 (indirect transition). The indirect bandgap values were calculated and presented in Fig. 3c. For Fe-1-asp sample the indirect bandgap of 2.07 eV is observed, showing the combined effect of goethite and ferrihydratite phases. It is clearly seen that on heating the samples, bandgap decreases as compared to the as-prepared sample with the red shift in absorption spectra. Phase transition from goethite to hematite along with the delocalization of electrons (while going from nano to comparatively bulk) results into the change of band structure, and hence the bandgap.

The BET surface area of the as-prepared nanorods calculated from the nitrogen adsorption/desorption isotherm was 86 m^2^/g. The heat treatment had a bearing on morphology as well as the surface area. The surface area is 89, 22 and 53 m^2^/g for Fe-1-250-Ar, Fe-1-450-Ar and Fe-1-450-Air samples respectively. From the SEM micrographs of these samples, the thickening of the individual nanorods also suggested the same effect.

The Raman spectra (Fig. 3d) of as-prepared sample Fe-1-asp, showed the well resolved peaks at 385, 299, 242, 479 and 552 cm^−1^ matching very well with the reported values for goethite structure^24^. The characteristic peaks of ferrihydrite are indistinguishable in the spectrum. The reason can be the low crystallinity and comparatively small fraction of the ferrihydrtie in the mixed phasic Fe-1-asp sample. XRD has suggested that on heating of these hydrated and hydroxylated as-prepared samples, they crystallize into hematite and meghamite phases. The Raman spectra of the heated samples are also in line with these observations. The TG recovered sample showed the sharp peaks of hematite phases at 222 (A_1g_), 245(E_g_), 289(E_g_), 296(E_g_), 402(E_g_), 493(A_1g_) and week peak at 610 (E_g_) cm^−1^^24^. As compare to this 900 ^o^C heated sample, rest of the samples showed broad nature of peaks due to their nano size. The size effect can also be monitored from the very small shifts in the peaks associated with the movements of iron ions (222, 245 and 298 cm^−1^) as compared to the remaining oxygen specific peaks.

### Husk like Iron Oxides

**Figure 4:**
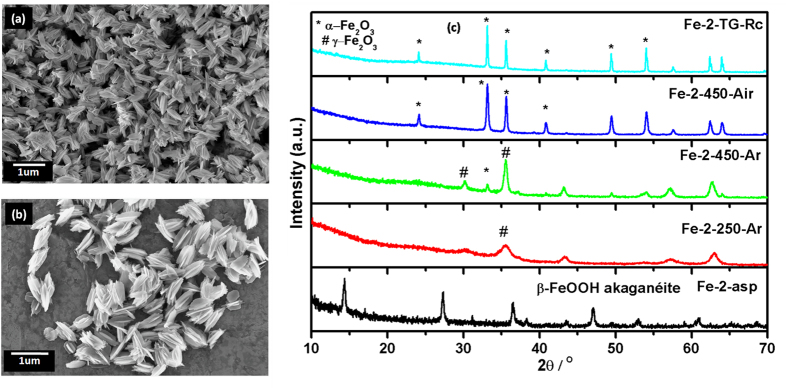
(**a**-**b**) SEM images of as-prepared husk like structure (Fe-2-asp) (**c**) XRD pattern of as-prepared and after different heat treatments.

MW-assisted hydrothermal treatment of Fe(II)oxalate yielded husk shaped iron hydroxides. The SEM images shown in Fig. 4(a-b) reflect that the as-synthesised material possess very unique morphology. The material crystallizes in the husk like structure with average dimensions of 803 nm (length) and 369 nm (width) with the thickness of individual chaff ~72 nm. To our best of knowledge, this type of morphology of iron oxide is not reported in literatures. The XRD pattern in Fig. 4c suggested that Fe-2-asp sample is pure β-FeOOH akaganéite phase with all the peaks matching very well with the corresponding standard pattern (PCPDF 34-1266).

Thermal behaviour of the as-prepared sample (SI-2a) revealed the two step weight loss. First weight loss of ~15% in the temperature range 100–300 ^o^C suggests the decomposition of residual organic moieties attached to particles along with the conversion of oxyhydroxide to oxide of iron. The second comparatively smaller weight loss of 1.93% can be attributed to structural arrangement of iron oxide. The corresponding DTA peaks also add to the information that the weight loss at the first stage is an endothermic process followed by second exothermic process. The residual sample, Fe-2-TG-Rc, was characterized by XRD and found to be consisting of α-Fe_2_O_3_ (Fig. 4c). On heating the as-prepared sample in argon atmosphere at 250 ^o^C for 4hr, single phasic β-FeOOH transforms into the γ-Fe_2_O_3_. It is clearly visible that from comparatively crystalline peaks of Fe-2-asp sample, on heating the material converted to the amorphous or poorly crystallized γ-Fe_2_O_3_ (Fe-2-250-Ar). However, peaks being broad and consisting of background features, it cannot be certainly called free from the α-Fe_2_O_3_ particles. In case of 450 ^o^C heated sample in argon atmosphere (Fe-2-450-Ar), additional peak at 33.22^o^ corresponding to the α-Fe_2_O_3_ starts appearing. On contrary to this, the sample heated in air atmosphere at same temperature (Fe-2-450-Air) is pure α-Fe_2_O_3_. SEM images of these two heat treated samples are given in SI-3. It is clear that at low temperature (250 ^o^C in Ar) there was no change in the morphology (SI-3a), however at moderately high temperature (450 ^o^C), the only change observed was thickening of the chaffs of this husk like structures (SI-3b). However, SEM image of 450 ^o^C heated in air sample (SI-3c) showed the similar thickening of chaffs to greater extent as compare to argon heated sample. In both the 450 ^o^C heated samples, the common feature of netting in some of chaffs was observed. Similar to the observation for the nanorods synthesized from iron sulphate, the SEM images of the sample after thermal analysis (SI-2a (inset)) showed that these particles are being diffused to form larger glassy clusters.

DRUV-Vis spectra of husk shaped iron oxides are presented in SI-2b. The spectrum of Fe-2-asp (akaganéite) samples was in accordance with that obtained for Fe-1-asp (goethite), both being the hydoxyoxide. The high energy transition peaks were positioned at same place but the low energy absorption bands near 640 and 900 nm of ligand field transitions of Fe^3+^ cation in octahedral environment were shifted, as compared to Fe-1-asp. The meghamite sample Fe-2-250-Ar, in comparison to other two samples, does not show the shoulder peak corresponding to the ligand-to-metal charge transfer (CT) i.e. the 6_t1u_ (O^2−^) → 2_t2g_ (Fe^3+^) transition which appears at 539 nm. The absorption peaks at 695 and 980 nm are also blue shifted. However, in case of the other two samples, these two peaks found to be diminishing, suggesting the increased particle size, which can be related to the thickening of the chaffs. The Tauc Mott equation as discussed in details for Fe-1 samples was used to calculate the indirect bandgap (SI-2c). The bandgap values varies with change in the phases. The as-prepared sample which was akaganéite showed the bandgap of 1.93 eV. The bandgap value decreased further with evaluation of hematite phase. However, this decrease in bandgap can be attributed to the thickening of the chaffs also. The 1.88 eV bandgap observed for the air heated sample is very close to the value reported for the bulk hematite. For this as-prepared husk like structure the specific surface area calculated was 22 m^2^/g, which is comparatively lesser than the surface area observed for nanorods. But when this sample was heated at 250 ^o^C the surface area increases to 78 m^2^/gm, suggesting the removal of the surface attached surfactant and further crystallization of phases. However, the further heating decreases the surface area value, more in argon atmosphere than in air. BET surface area values were 15 and 21 m^2^/g respectively for Fe-2-450-Ar and Fe-2-450-Air, respectively.

Though XRD fails to easily distinguish between the isostructural forms meghamite and magnetite, Raman provides an efficient way to do so. However, in present case, where samples are less crystalline and in nano regime, the characteristic peaks occur at frequency lower than 650 cm^−1^ (SI-2d). The 250 ^o^C heated sample, which XRD showed to be pure meghamite, showed the characteristic but very small Raman features at 350 and 730 cm^−1^, along with the comparatively more intense hematite peaks. Which further suggests that the sample Fe-2-250-Ar is also mixed phasic. The other heat treated samples showed the highly intense characteristic Raman peaks of hematite similar to the heated nanorod samples.

### Distorted Cubes of Iron Oxides

**Figure 5:**
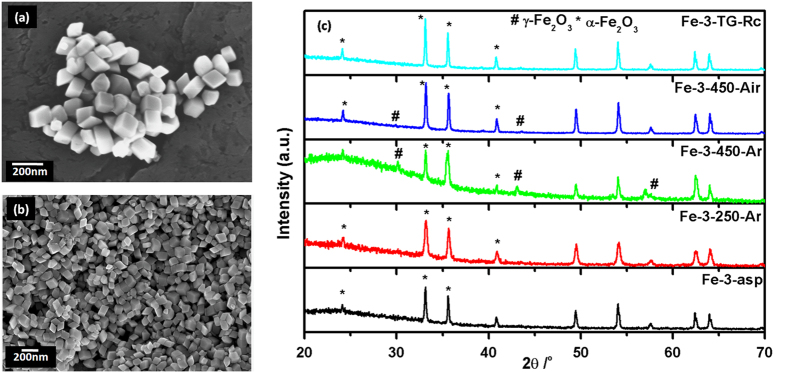
(**a**-**b**) SEM of as-prepared distorted cubes (Fe-3-asp) (**c**) XRD pattern of distorted cubes; as-prepared and after different heat treatments.

The SEM images of as-prepared sample from FeCl_3_.6H_2_O as precursor are shown in Fig. 5(a-b). The sample consists of mostly distorted cubes along with the very small fraction of secondary shapes. The estimated average size as visible from SEM is ~120 nm. On phase characterization using XRD, the sample revealed to be consisting of pure α-Fe_2_O_3_ (Fig. 5c). The peaks matches well with the reported standard pattern of α-Fe_2_O_3_ (PCPDF 33-0664).

On thermal analysis, only a very small change in the weight was observed (SI-4a). α-Fe_2_O_3_ being comparatively stable than its oxyhydroxide and ferrihydrite counterparts, only phase transition from alpha to gamma is possible. The small change observed can also be attributed to the loss of the remaining organic component. The residue obtained (Fe-3-TG-Rc) reflected to be pure α-Fe_2_O_3_ (Fig. 5c), same as the as-prepared sample. Morphological characterization of the different heated samples; Fe-3-250-Ar, Fe-3-450-Ar and Fe-3-450-Air is shown in SI-5. From SEM images, it is evident that on heating the sample at 250 ^o^C in inert atmosphere, it retained its characteristic morphology. However, on heating the sample at 450 ^o^C both in inert and air atmosphere, the particles further distort and tends to convert into the energetically more stable spherical particles with retention of original particle size. Phase evaluation of these heated samples as shown in Fig. 5c, reflects that although all the samples are α-Fe_2_O_3_, small peaks comprising of γ-Fe_2_O_3_ are also observed, mainly in 450 ^o^C heated sample. The 450 ^o^C argon heated sample (Fe-3-450-Ar) contained the more amount of γ-Fe_2_O_3_ as compared to air heated sample. The high intensity peak of γ-Fe_2_O_3_ overlaps with the peaks of α-Fe_2_O_3_ and thus increase in the intensity is observed.

The absorption spectra (SI-4b) showed the characteristic features of Fe^3+^ ion in octahedral environment, as mentioned for earlier samples. The DRS spectrum was used to calculate the indirect bandgap (SI-4c). The bandgap of Fe-3-asp 1.98 eV decreases on heating the sample at 250 ^o^C in air. Air heated sample at 450 ^o^C, in which hematite is in lower concentration than the argon heated sample, showed further reduced bandgap. The micron sized cubes obtained has the surface area of 13 m^2^/g. For the heated samples, Fe-3-250-Ar, Fe-3-450-Ar and Fe-3-450-Air, the surface area measured are 17, 11 and 10 m^2^/g, respectively. All the samples being micron sized and highly crystalline with hematite as the major phase, no features other than that belonging to characteristic hematite are observed (SI-4d).

### Nano Cubes of Iron Oxides

**Figure 6:**
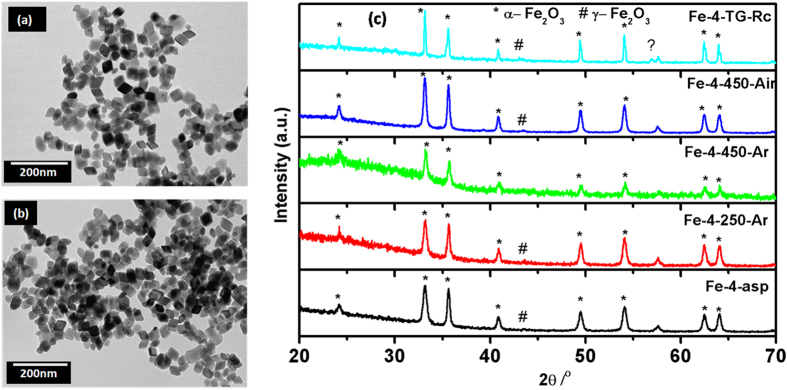
(**a**-**b**) TEM images of nano cubes (Fe-4-asp) (**c**) XRD pattern of nano cubes; as-prepared and after different heat treatments.

When Fe(III)nitrate nonahydrate was used as the iron precursor cube shaped iron oxide is obtained. Contrary to the cubes obtained from the FeCl_3._6H_2_O precursor, these cubes are smaller with the size of 20 nm as observed by the TEM images (Fig. 6a-b). In XRD pattern (Fig. 6c), the majority phase found to be α-Fe_2_O_3_, with small contribution of γ-Fe_2_O_3_.

The corresponding XRD peaks were very well matched with the standard pattern of α-Fe_2_O_3_ (PCPDF 33-0664). The as-prepared sample, Fe-4-asp, was analysed for its thermal nature by TG/DTA. Owing to the presence of well crystallized iron oxide in as-prepared sample (Fe-4-asp), feeble change in the corresponding weight was expected. However, the small weight loss of 1.5% in the temperature range of 150–500 ^o^C (SI-6a) attributable to the decomposition of the residual surfactant is observed. The XRD pattern of TG/DTA analysed sample (Fe-4-900-TG-Rc), as shown in Fig. 5c, has the same composition as that of Fe-4-asp. Similarly, the heated samples; Fe-4-250-Ar, Fe-4-450-Ar and Fe-4-450-Air consist of α-Fe_2_O_3_ along with small and varying amount of γ-Fe_2_O_3_. With increasing the temperature the peak width of the corresponding phases decreases, suggesting the increase in average crystallite size. The shape and morphological characterization using SEM (SI-7) suggested that the heat treatments play significant role in alteration of morphology. As-prepared sample, Fe-4-asp, consisted of majority of particles with the sharp cubic geometry. However, on heating the particles get slightly deformed.

The DRUV-Vis absorption spectra of as-prepared as well as heat treated samples are shown in SI-6b. The spectra showed characteristic strong transitions in UV range along with the week visible light transitions. The characteristic transitions of Fe^3+^ in octahedral environment, including ligand to metal charge transfer (250–400 nm), pair excitation process (400–600 nm) of magnetically coupled Fe^3+^ ions and two strong absorption bands near 640 nm of ligand field transitions of Fe^3+^ cation are diffused and overlapped.

The bandgap values calculated, considering the indirect band structure, were 1.85, 1.83, 1.68 and 1.61 eV for Fe-4-asp, Fe-4-250-Ar, Fe-4-450-Ar and Fe-4-450-Air samples, respectively (SI-6c). The surface area of these as-prepared nano cubic iron oxide was 37.24 m^2^/g and greater than that observed for the micron sized cubes. As was observed from the SEM micrographs, the heat treatment led to the diffusion of nano cubes into micron cubes which had direct influence in the surface area. The surface area for Fe-4-250-Ar, Fe-4-450-Ar and Fe-4-450-Air, are 29, 17 and 16 m^2^/g respectively. From XRD pattern all the samples consisted of hematite phase with very small impurity from meghamite, the Raman spectra also agrees with this trend with no features other than hematite was observed (SI-6d).

### Porous Spheres of Iron Oxides

**Figure 7:**
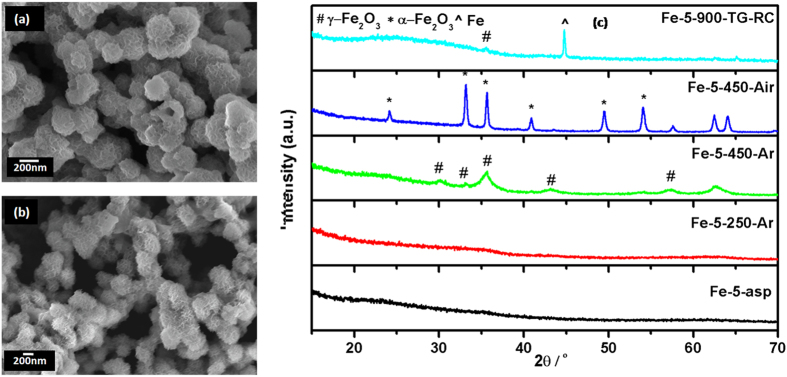
(**a-b**) SEM images of as-prepared porous spheres (Fe-5-asp) (**c**) XRD pattern of porous sphere; as-prepared and after different heat treatments.

MW-assisted hydrothermal heating of Fe(II) D-gluconate dehydrate yielded dark black colored product. However, after subsequent washings with water and alcohol, the color quickly turns to greenish yellow. The as-prepared sample (Fe-5-asp) showed almost spherical porous structures (Fig. 7a-b). Some of the incompletely grown and broken spherical particles suggest that these particles could be hollow at the core. However, these spherical porous particles are agglomerated and could not be separated even after several modifications in reaction conditions (temperature, time, stoichiometry etc.). The average size obtained from SEM micrographs was ~150 nm, with a small inhomogeneity in the shape and size. The XRD pattern (Fig. 7c) revealed the amorphous nature of Fe-5-asp sample.

Thermal behaviour of this amorphous sample as studied by TGA/DTA (SI-8a) showed the continuous weight loss (37%) till 400 ^o^C followed by the stepped weight change (20%) at 644 ^o^C. The continuous weight loss can be attributed to the loss of absorbed water, organic species and crystallization to the oxide. The XRD of the residue obtained (Fe-5-TG-Rc) showed that the as-prepared amorphous material crystallizes into Fe-metal along with the small amount of γ-Fe_2_O_3_. This conversion of amorphous product into crystallized metal rather than oxide form indicated that the condition in the thermal analysis i.e. nitrogen atmosphere and 900 ^o^C is reducing, sufficient for the conversion of as-prepared amorphous sample into metallic product. The endothermic peak observed at the 644 ^o^C with 20% mass change in DTA curve is attributable to the transformation of α-Fe_2_O_3_ to Fe metal. The XRD pattern of different heated samples Fe-5-250-Ar, Fe-5-450-Ar and Fe-5-450-Air are also shown in Fig. 6c. The as-prepared sample was amorphous as mentioned above. The argon atmosphere heated sample at 250 ^o^C retains the amorphous nature, however as the heating temperature increases from 250 to 450 ^o^C the crystallization starts and the peaks corresponding to the γ-Fe_2_O_3_ (PCPDF 39-1346) appears. The same heat treatment in air resulted in the pure α-Fe_2_O_3_ (PCPDF 33-0664), as indicated in the XRD pattern. The effects of these heat treatments on the shape of the sample were also prominent, as visible from the SEM micrographs (SI-9). When the samples were heated at 250 ^o^C, the particles look more porous (SI-9a), suggesting the removal of organic structure from the surface. As the heating temperature increased the thickness of the porous structure also increased. In case of air heated sample (SI-9c), the increase in thickness is more prominent as compare to the argon heated sample.

DRUV-Vis spectra of these porous spheres are presented in SI-8b. Comparative blue shift of the as-prepared spectrum also confirms the non-crystalline nature of sample, with absence of sharp characteristic features of three known transitions. The clear observance of 535 nm peak in air heated sample further confirms the full conversion of amorphous to crystalline, γ-Fe_2_O_3_ to α-Fe_2_O_3_, as product. The indirect bandgap values calculated for the amorphous materials (SI-8c) suggests the probability of amorphous α-Fe_2_O_3_. The BET surface area was found to be 36, 19, 150 and 38 m^2^/g for Fe-5-asp, Fe-5-250-Ar, Fe-5-450-Ar and Fe-5-450-Air, respectively. The sudden increased surface area of Fe-5-450-Ar is due to the crystallization along with the complete removal of surfactant molecules. The Raman spectra (SI-8d) of these very interesting samples collaborate with the structural data obtained from the XRD pattern. The XRD data suggested the absence of characteristic peaks for oxide system, especially Fe-5-asp and Fe-5-250-Ar, indicating the absence of long range ordering, so does the Raman data. The amorphous as-prepared and 250 ^o^C heated porous spheres did not show any characteristic Raman features. Further heat treatment resulted in less crystalline meghamite, and hence the features of meghamite were also not observed in Raman spectra. Even features of hematite were also not observed suggesting sample consisting of the purely meghamite phase. The metallic phase of iron obtained after the TG/DTA analysis in inert atmosphere till 900 ^o^C found to be consisting of small amount of meghamite and not hematite. Due to the higher sensitivity of Raman as compare to the XRD, the existence of meghamite, though in very small amount, was clear.

### Self-oriented Flowers of Iron Oxides

**Figure 8:**
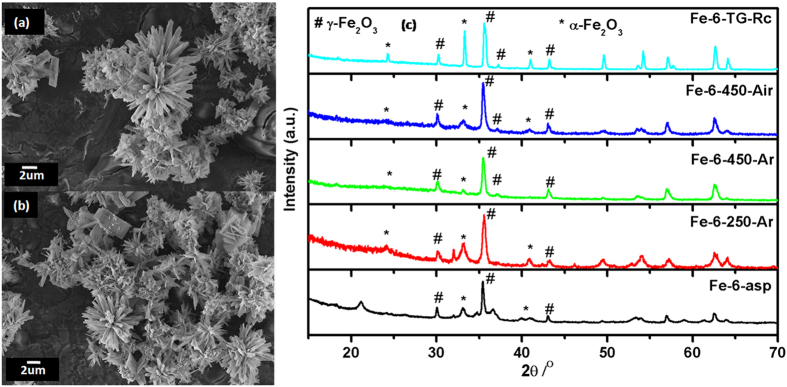
(**a-b**) SEM images of as-prepared self-oriented flowers (Fe-6-asp) (**c**) XRD pattern of self-oriented flowers; asp and after different heat treatments.

The product obtained from Fe(0) pentacarbonyl as precursor consisted of self-oriented flower like structure. On careful examination of their SEM images (Fig. 8(a-b)), it was observed that the nano-sheets were getting rearranged to form the flower like structure. The average diameter of these self-oriented flower like structure is 250–300 nm. Along with the major self-oriented flower like morphology, secondary morphologies like plates and agglomerated particles were also observed, though in very less quantity. The phase characterization of the as-prepared sample (Fe-6-asp) revealed that the sample consists of γ-Fe_2_O_3_ as a major phase along with hematite and other hydroxides (Fig. 8c). Thermal behaviour of this sample, as observed in SI-10a, comprises of large weight loss (13%) in the range of 125 to 500 ^o^C due to loss of organic template. An endothermic peak at 700 ^o^C without change in the mass suggests that there is a structural change involved. The SEM micrograph of this thermally analysed sample is shown in SI-10a inset, which reflects the diffused morphology of the material.

When Fe-6-asp was heated in different atmosphere at different temperatures, the distinguishable effect on morphology and phase was observed. SEM images of 250 ^o^C heated sample (SI-11a) showed that, the plate/petals of the flowers which are nothing but the sheets, are getting pierced with increasing the temperature which is more prominent in case of 450 ^o^C heated sample (SI-11b). Fe-6-450-Ar (SI-11c) showed the complete degradation of the petals into nano sized agglomerated particles. Phase characterization using XRD (Fig. 8c) revealed that with increasing temperature α-Fe_2_O_3_ characteristic peaks starts appearing (PCPDF 33-0664). Thermal analysed sample Fe-6-TG-Rc found to be mixture of α and γ-Fe_2_O_3_.

The electronic absorption spectra of the as-prepared as well as heated samples are shown in SI-10b. The absorption spectra consist of the all three characteristic absorption ranges as explained for the Fe-1 sample. In comparison to the heat treated samples the as-prepared sample Fe-6-asp did not show the shoulder peak at 535 nm. The peak at 535 nm is characteristic of the α-Fe_2_O_3_, where the double excitation of the Fe^3+^ ion occurs in octahedral environment. The indirect bandgap of Fe-6-asp sample was found to be 1.69 eV, which further decreases with increasing temperature, due to change in the concentration of hematite. The indirect bandgap of the 450 ^o^C air heated sample was found to be very well matching with the reported value of ~2 eV of hematite. However, the XRD pattern suggests that hematite concentration was relatively same in all three heated samples. It can be assumed that heat treatment in different atmosphere have different effect of the surface defect and hence change in bandgap values. The surface area of as-prepared self-oriented flowers is 88 m^2^/g. The BET surface area values for heated samples are 53, 37 and 26 m^2^/g for Fe-6-250-Ar, Fe-6-450-Ar and Fe-6-450-Air, respectively. The decreasing trend in the values is attributable to the fragmenting of the flower leaves and crystallization. The Raman spectra of these samples showed broad and small peaks of hematite but no features of meghamite and megnatite were observed, may be due to less crystallinity. In the 250 ^o^C heated sample (Fe-6-250-Ar), the broad hump around 500–515 cm^−1^ region and 650–700 cm^−1^ region suggests the presence of both meghamite and magnetite in the sample along with hematite (SI-10d).

The synthesis of shaped iron oxide nanoparticles by using this comparatively greener and sustainable method gave different morphologies on mere change of the starting precursor. In a recent article, Pileni has explained the different possible mechanism for the variable morphology formation^25^. We also believe that, the inorganic anions from the precursor itself get selectively adsorbed on some of facets during the crystallization and thus direct the final morphology. In present case also, the evolution of different morphology could be associated with anions of starting iron salts, which were different in each case; SO_4_^2−^, C_2_O_4_^2−^, Cl^−^, NO^3−^, C_6_H_11_O_7_^−^ and carbonyl groups of Fe(II) sulphate heptahydrate, Fe(II) oxalate dehydrate, Fe(III) chloride hexahydrate, Fe(III) nitrate nonahydrate, Fe(II) D-gluconate dehydrate and Fe(0) pentacarbonyl respectively, which may act as facet coating agent during crystallization process^25^. We also feel that CTAB based micelles can also act as template/structure directing in case of spherical shaped iron oxides. It can also be the combination of both anion facet coating and template/structure directing. However, the exact mechanism for the evolved morphologies could not be traced down. The solubility of starting materials also affects the kinetic of the reaction, hence also the crystallization of specific phases. In present case, the iron precursors used are of different solubility specifically in water. The solubility values being; 28.8, 0.008, 91.8, 138, 10 and 5-10 g in 100 ml of water at room temperature for Fe(II) sulphate heptahydrate, Fe(II) oxalate dehydrate, Fe(III) chloride hexahydrate, Fe(III) nitrate nonahydrate, Fe(II) D-gluconate dehydrate and Fe(0) pentacarbonyl respectively. The highly soluble salts, Fe(III) chloride hexahydrate and Fe(III) nitrate nonahydrate, resulted into the formation of most stable crystalline α-Fe_2_O_3_ as major phase. Remaining comparatively less soluble precursors led to the formation of mixed phases of hydroxides. The amorphous nature of product obtained from Fe(II) D-gluconate dehydrate as described earlier in spite of sufficient solubility is attributable to its bulkier leaving group. The gluconate group require larger energy to decompose and hence major fraction of the heat supplied may be utilized in the breaking down of precursor itself, leaving no energy for the crystallization. The samples thus synthesized showed surface area ranging from 13 to 88 m^2^/gm, for Fe-3-asp (distorted cubes) to Fe-6-asp (porous sphere). Further, these surface area values get affected by the subsequent heat treatment due to increasing diameters and removal of the defects, as shown in Table. 2. Similarly, the bandgap values were also found to be affected by the crystallizing phases along with the feeble contribution of respective size. The bandgap values for all the 450 ^o^C heated samples in argon found to be near the value reported for the α-Fe_2_O_3_ (2.0 eV).

Based on these results, it is clear that along with the different morphologies the starting precursor material also affects the phases of the material. The different phases obtained from the different precursor along with the morphologies are summarized in Table 1. Crystallization of hematite or meghamite and their ratios in the samples is very much dependent on the phase and crystallinity of as-prepared sample, which itself is the consequence of solubility of precursors used. In case of the nanorods obtained from the Fe(II)sulphate heptahydrate the as-prepared sample is a mixture of goethite and ferrihydrite which eventually get converted into mixed phases of meghamite and hematite with varying ratios. The akaganéite nano husks obtained from the Fe(II)oxalate dehydrate transforms to pure meghamite structure at 250 ^o^C in argon atmosphere. On further heating, peaks characteristic of the hematite was also observed. However, when the as-prepared sample obtained was hematite it remains hematite at 250 ^o^C and only at 450 ^o^C meghamite starts appearing. In case of amorphous product obtained from the Fe(II)D gluconate, the porous spheres remained amorphous at 250 ^o^C, which on further heating at 450 ^o^C gets converted to the pure meghamite in argon and pure hematite in air atmosphere. Whereas, the residual samples from TG/DTA of these spheres, showed formation of pure Fe metal, along with the meghamite in small fraction. For the major meghamite phasic flowers obtained from the iron pentacarbonyl, further heat treatment gave mixtures of hematite and meghamite. The effect of heating on different morphologies at relatively low temperature i.e. 250 ^o^C was indistinguishable. However, the 450 ^o^C heat treatments showed observable changes, in shape and morphology of different as-prepared iron oxides.

### Magnetic Studies of Shaped Iron Oxides

**Figure 9:**
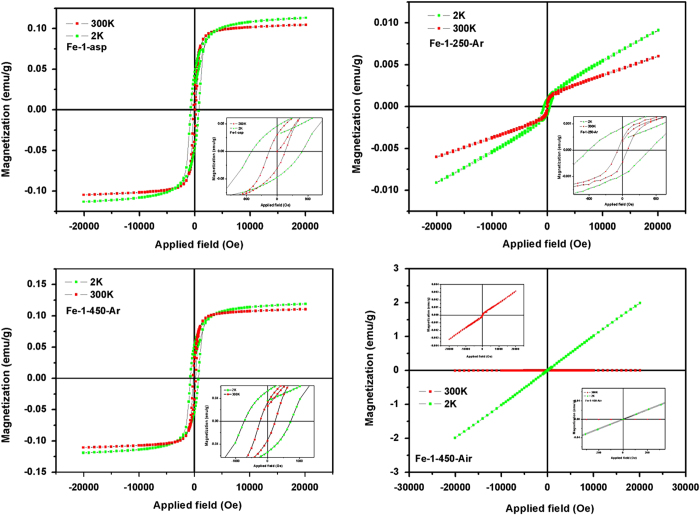
Field dependent magnetization of nanorod samples at 2 K and 300 K.

Iron based hydroxides and oxides show interesting magnetic properties which are highly influenced by their phases, shape and size. Ferrihydrites behaves antiferromagnetically below 120 K however ferromagnetism arises due to uncompensated spins at or inside of the particles^26^. Similarly goethite is expected to behave antiferromagnetically at about 400 K^27^. The hematite undergoes two type of magnetic transitions at temperatures 950 K and 260 K, known as Neel Temperature (T_N_) and Morin temperature respectively (T_M_)^28^. Below T_N_, it shows week ferromagnetism and at T_M_ weak ferromagnetic to antiferromagnetic transition occurs. In present study, different as-prepared as well as heat treated iron hydroxides/oxides, samples were analyzed for their magnetic properties by measuring their field dependent magnetization in the range of −2T to 2T, below and above T_M_ i.e. 2 K and 300 K. The representative field dependent magnetization of nanorod samples (Fe-1) are shown in Fig. 9.

For the as-prepared nanorods, consisting of goethite and ferrihydrite as the major phases, antiferromagnetism was expected. However, week ferromagnetism is observed at room temperature with saturation magnetization (M_s_) 0.104 emu/g, which increases to 0.113 emu/g, on decreasing the measurement temperature (2 K). This unusual magnetic behaviour is attributable to the uncompensated magnetic saturation of spins originated due to reduced particle size or the surface charges. When the temperature was 2 K, the increase in coercivity (H_c_) was also observed as compare to the 300 K hysteresis loop. The enhanced exchange between the randomly frozen spins at such a low temperature results into increase of H_c_ from 231.95 to 730.83 Oe. Remanent magnetization (M_r_) values also increases from 0.0294 to 0.045 emu/g. For the Fe-1-250-Ar sample, the change in hysteresis loop shape was drastic. As mentioned above the ferromagnetism with reasonable saturation magnetization value could be easily linked to the uncompensated spins or defect at the surface.

The heat treatment at 250 ^o^C results into the removal of such defect and crystallization of α and γ morphs of Fe_2_O_3_, in different ratios. The combined effect of antiferromagnetsim of α-Fe_2_O_3_ and superparamagnetism of γ-Fe_2_O_3_ results into the magnetic behaviour with M_s_ ~ 0.006 and M_r_ ~ 4.38 × 10^−4^ emu/g at room temperature, 300 K. At 2 K measurements, the M_s_ increases to 0.009 and M_r_ to 8.7 × 10^−4^ emu/g. H_c_ values at both the temperature decreases drastically as compared to the Fe-1-asp sample. For the Fe-1-450-Ar the enhanced magnetism was observed, with distinct ferromagnetism feature, assigned to the increased heamatite phases over meghamite phase, with magnetic parameters similar to that observed for the Fe-1-asp sample. However, for the air heated sample which consists of thick rods of highly crystalline hematite and meghamite phases, Fe-1-450-Air the magnetic behaviour changed completely. At room temperature, 300 K, the hematite phase predominates with the anitferromagntic nature, while at low temperature 2 K, the superparamagnetic behaviour, characteristic of meghamite phase near zero value of M_r_ and H_c_ was observed.

For the nanohusk like structure, for the as-prepared sample which mainly consists of β- akaganéite phase, the room temperature behaviour was near paramagnetic with no saturation and zero M_r_ and H_c_ values (SI-12). However at low temperature the sample behaved weekly ferromagnetically. For the same morphology, the heated samples in argon atmosphere, Fe-2-250-Ar and Fe-2-450-Ar, showed week ferromagnetism but with increased M_s_ (The values are tabulated in SI-Table 1). The argon heated samples being mixed with meghamite phases showed the complex magnetic nature due to contribution from the individual phases. In case of Fe-2-450-Air, the single phasic hematite, the week ferromagnetism at 300 K gets converted to the behaviour which can be assigned to the antiferromagnetism of hematite phase.

The as-prepared micron sized hematite cubes, at 300 K measurements showed antiferromagnetism as expected for the hematite particles (SI-13). However in 2 K hysteresis loop, sample shows very small amount of hysteresis characteristics of uncompensated charges at such low temperature. In case of as-prepared sample no saturation was reached, till the measurement field, 2 T. No saturation was also observed for the 250 ^o^C heated sample. The Fe-3-450-Ar sample showed comparatively more ferromagnetic nature with M_s_ values 0.119 emu/g at RT and 0.0169 emu/g at 2 K. While comparing the magnetic behaviour of the two cubic structure obtained, Fe-3-asp (micron sized cubes) and Fe-4-asp (nano cubes), the nanocubes containing meghamite as one of the phase, showed near superparamagnetic behaviour at both the temperatures, 2 K and 300 K (SI-14). The meghamite particles are known to be superparamagnetic below 12 nm particles size. However, this magnetic nature gets overpassed by the presence of lager particles as seen in the magnetic measurements of heated samples.

The as-prepared porous spheres (Fe-5-asp) showed near superparamagnetism (SI-14) at RT with no M_s_ and zero H_c_ and M_r_ values (SI-Table 2). However, on decrease in temperature to 2 K, the week ferromagnetism was observed, which can be attributed to the surface structure of porous spheres. Similar trend for the Fe-5-250-Ar sample was observed, with change in slope for the 300 K hysteresis. For these two samples, Fe-5-asp and Fe-5-250-Ar, the magnetization could not be assigned to specific phases. The 450 ^o^C heated samples (Fe-5-450-Ar) consisting of nano-meghamite phase showed the superparamagnetism with the M_s_ ~ 0.387 emu/g at 300 K. The 2 K hysteresis loop suggests the increased interaction of frozen spins at the surfaces. When the sample was heated in air (Fe-5-450-Air), giving the single phase hematite particles which are known to be magnetically inactive phase, the paramagnetism was completely removed. But the small amount of the ferromagnetism was retained in the system.

The Fe-6-asp sample being mixed phases of oxides and hydroxide, with self-oriented flowers as the major morphology along with the nanoparticles, showed the combined effect of phases and morphology in a ferromagnetic hysteresis loop (Figure SI-15) with M_s_ magnetisation value increasing from 0.40 to 0.65 to 1.94 emu/g at 300 K. However the air annealed sample showed decreased magnetization at 300 K and also the week ferromagnetism turns to the superparamagnetism. From XRD pattern of these two samples, Fe-6-450-Ar and Fe-6-450-Air, the ratio of meghamite and hematite was almost constant. However the drastic change in magnetism was observed, similarly from all of the above samples discussed. It is known that the M_s_, H_c_ and M_r_ values are highly dependent on annealing conditions leading to change in different surface properties along with change in phases. Here, when the samples were heated in argon atmosphere the surface defects were retained into the morphology, contributing to the magnetism. However, heat treatment in air results into the complete removal of defect structures from the surface and hence the properties obtained were majorly phase dependent.

## Conclusions

Efficient and easy to implement solvo-thermal protocol for the synthesis of range of shaped iron oxides was developed. Importantly, by simply changing the precursors iron salts, we were able to change the shape of irons oxides, using exactly same reactions conditions. Several unique shapes of iron oxides were discovered. Comprehensive characterization of as-synthesised materials using electron microscopy, diffraction techniques, spectrophotometry and spectrometry led to the inviolable insight about the basic and fundamental properties of these synthesized iron oxides. In view of their phase and morphology dependent magnetic properties, detailed magnetic measurements were performed and the correlation of data also helped to further confirm the structure, phase and property relationship.

## Methods

All the synthesis were carried out in Ethos-1 MW reactor using PRO-24 high throughput rotor when reaction mixture volume was below 70 ml and for higher reaction mixture volume Ethos-1 Lit rotor was used.

### Synthesis of Nanorods of Iron Oxides

3 g (0.0499 mol) of Urea and 5 g (0.0137 mol) CTAB were dissolved in 250 ml deionised water by stirring at 500 rpm in 1 litre reactor for 15 min. 250 ml cyclohexane was added to the above solution followed by 15 ml of 1-pentanol. The reaction was further stirred for 15 min. Iron precursor FeSO_4_.7H_2_O, 2.780 g (0.01 M) was added to the solution and mixed for another 30 min. The reactor was tightly closed and was exposed to MW radiation (800 W maximum power) at 80 ^o^C (with 30 min ramp) for 4 h, under stirring.

### Synthesis of Husk like Iron Oxides

Similar to above synthesis, but by using (0.01 mol) Fe(II)oxalate dehydrate. The condition for the synthesis was 80 ^o^C (with ramp time of 30 min) for 30 min reaction time.

### Synthesis of Distorted Iron Oxides

0.3 g (0.00499 mol) of Urea and 0.5 g (0.00137 mol) CTAB were dissolved in 25 ml deionised water by stirring at 500 rpm in a 100 ml beaker for 15 min. 25 ml cyclohexane was added to the above solution followed by 1.5 ml of 1-pentanol. The reaction was further stirred for 15 min. Iron precursor FeCl_3_.6H_2_O, 0.2703 g (0.001 mol) was added to the solution and mixed for another 30 min. The reaction mixture was transferred to 70 ml Teflon reactor. The reactor was tightly closed and kept in microwave for the microwave assisted heat treatment. The condition for the reaction was 160 ^o^C (with ramp time of 30 min) for 2 h.

### Synthesis of Nano Cubes of Iron Oxides

Similar to synthesis of nano-rod like shape, but by using (0.001 mol) Fe(III)nitrate nonahydrate. The condition for the reaction was 160 ^o^C (with ramp time of 30 min) for 4 h with stirring.

### Synthesis of Porous Spheres of Iron Oxides

0.15 g (0.00249 mol) of Urea and 0.5 g (0.00137 mol) CTAB were dissolved in 25 ml deionised water by stirring at 500 rpm in a beaker for 15 min. 25 ml cyclohexane was added to the above solution followed by 1.5 ml of 1-pentanol. The reaction was further stirred for 15 min. Iron precursor Fe(II) D-gluconate dehydrate, 0.4821 g (0.001 mol) was added to the solution and mixed for another 30 min. The reaction mixture was transferred to 70 ml teflon reactor. The reactor was tightly closed and kept in microwave for the microwave assisted heat treatment. The condition for the reaction was 180 ^o^C (with ramp time of 30 min) for 30 min.

### Synthesis of Self-oriented Flowers of Iron Oxides

3.0 g (0.0499 mol) of Urea and 5 g (0.0137 mol) CTAB were dissolved in 250 ml deionised water by stirring at 500 rpm in 1 litre reactor for 15 min. 250 ml cyclohexane was added to the above solution followed by 15 ml of 1-pentanol. The reaction was further stirred for 15 min. 1 ml (0.0005 mol) Fe(0) pentacarbonyl was added to the solution and mixed for another 30 min. The reactor was tightly closed and kept in microwave for the microwave assisted heat treatment. The condition for the reaction was 140 ^o^C (with ramp time of 30 min) for 30 min with simultaneous stirring at 90%.

In all synthesis, after the completion of reaction, the mixture was cooled to room temperature, the precipitated products were isolated by centrifugation; washed thoroughly, by water and ethanol and then the product was air dried.

### Characterization techniques

For the morphological characterization and particle size distribution, their micrographs were recorded on scanning electron microscope using Zeiss Ultra FEG 55 operated at 5 kV. TEM images of nano iron oxide were recorded on ZEISS LIBRA120 EFTEM. The crystallographic phases of the prepared iron samples were investigated using a Panalytical X’Pert Pro powder X-ray diffractometer (XRD) using Cu-K*α* radiation. The thermal gravimetric analysis curve was recorded on a NETZSCH STA 449C TG thermal analyzer from 25 to 900 °C at a heating rate of 10 ^o^C min^−1^ in nitrogen. The surface area and pore size distribution were obtained using the Brunauer-Emmet-Teller (BET) from N_2_ physisorption data at 77 K recorded using a Micromeritics ASAP 2020 analyzer. About 50 mg of each sample was degassed at 10^−6^ Torr and 120 ^o^C prior to N_2_ adsorption. Diffuse reflectance UV-visible spectra were recorded on a JASCOV-670 spectrophotometer, using BaSO_4_ as a reference. Raman scattering measurements are performed in the backscattering geometry using a triple grating Raman spectrometer (T64000: Jobin Yvon) equipped with a liquid nitrogen cooled charge-coupled device. The excitation source is the 647.1 nm line of a mixed gas laser (Stabilite 2018: Spectra Physics). The DC magnetization was recorded using Quantum Design (QD) Inc. Superconducting Quantum Interference Device (SQUID) magnetometers (Models MPMS-5 and SQUID-VSM).

## Author Contributions

V. P. generated the idea and planned the project. F. N. S. carried out the experiments. Both the authors participated in data analysis. V. P. and F. N. S. wrote the manuscripts.

## Additional Information

**How to cite this article**: Sayed, F. N. and Polshettiwar, V. Facile and Sustainable Synthesis of Shaped Iron Oxide Nanoparticles: Effect of Iron Precursor Salts on the Shapes of Iron Oxide. *Sci. Rep*. 5, 09733; doi: 10.1038/srep09733 (2015).

## Supplementary Material

Supplementary Information

## Figures and Tables

**Figure 1:**
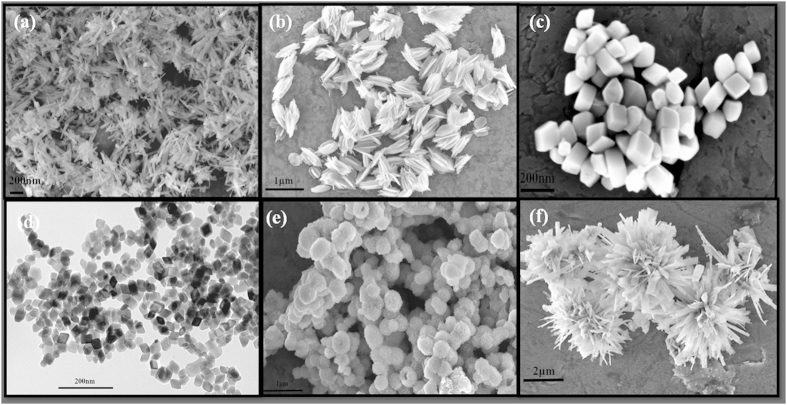
SEM images of shaped iron oxides with six different shapes (**a**) Nanorod, (**b**) Nanohusk, (**c**) Distordted cubes, (**d**) Nanocubes, (**e**) Porous spheres and (**f**) Self-oriented flowers.

**Table 1:** Shape and phase evaluation of different iron oxides.

Precursor	Shape	As-prepared samples	heated 250 °C in argon	heated 450 ^o^C in argon	heated 450 ^o^C in air	TG/DTA recovered sample
**Fe(II)sulphate heptahydrate**	nanorods	α-FeOOH + 5Fe_2_O_3_.9H_2_O	α-Fe_2_O_3_ + γ-Fe_2_O_3_	α-Fe_2_O_3_ + γ-Fe_2_O_3_	α-Fe_2_O_3_ + γ-Fe_2_O_3_	α-Fe_2_O_3_
**Fe(II)oxalate dihydrate**	nano husks	β-FeOOH	γ-Fe_2_O_3_	α-Fe_2_O_3_ + γ-Fe_2_O_3_	α-Fe_2_O_3_	α-Fe_2_O_3_
**Fe(III)chloride hexahydrate**	cubes	α-Fe_2_O_3_	α-Fe_2_O_3_	α-Fe_2_O_3_ + γ-Fe_2_O_3_	α-Fe_2_O_3_ + γ-Fe_2_O_3_	α-Fe_2_O_3_
**Fe(III)nitrate nonahydrate**	nanocubes	α-Fe_2_O_3_ + γ-Fe_2_O_3_	α-Fe_2_O_3_ + γ-Fe_2_O_3_	α-Fe_2_O_3_	α-Fe_2_O_3_ + γ-Fe_2_O_3_	α-Fe_2_O_3_ + γ-Fe_2_O_3_
**Fe(II)D gluconate dihydrate**	porous spheres	amorphous	amorphous	γ-Fe_2_O_3_	α-Fe_2_O_3_	Fe + γ-Fe_2_O_3_
**Fe(0)pentacarbonyl**	self arranged flowers	γ-Fe_2_O_3_ + oxyhydroxides	α-Fe_2_O_3_ + γ-Fe_2_O_3_	α-Fe_2_O_3_ + γ-Fe_2_O_3_	α-Fe_2_O_3_ + γ-Fe_2_O_3_	α-Fe_2_O_3_ + γ-Fe_2_O_3_

**Table 2:** Textural properties and bandgap of shaped iron oxides.

Precursor	Shape	As-prepared samples		Heated 250 ^o^C in argon		Heated 450 ^o^C in argon		Heated 450 ^o^C in air	
		Surface Area (m^2^/gm)	Bandgap (eV)	Surface Area (m^2^/gm)	Bandgap (eV)	Surface Area (m^2^/gm)	Bandgap (eV)	Surface Area (m^2^/gm)	Bandgap (eV)
**Fe(II)sulphate heptahydrate**	nanorods	86	2.07	89	1.84	22	1.84	53	1.84
**Fe(II)oxalate dihydrate**	nano husks	22	1.93	78	1.71	15	1.26	21	1.88
**Fe(III)chloride hexahydrate**	cubes	13	1.98	17	1.54	11	1.66	10	1.97
**Fe(III)nitrate nonahydrate**	nanocubes	37	1.83	29	1.61	17	1.68	16	1.85
**Fe(II)D gluconate dihydrate**	porous spheres	36	1.80	19	1.33	150	1.01	38	1.82
**Fe(0) pentacarbonyl**	self arranged flowers	88	1.56	53	1.83	37	1.05	26	1.69
