# Supplementary material for: Facile and Sustainable Synthesis of Shaped Iron Oxide Nanoparticles: Effect of Iron Precursor Salts on the Shapes of Iron Oxides
Source: Sci Rep. 2015 May 5;5:9733. doi: 10.1038/srep09733 (PMC4419518; doi:10.1038/srep09733)
Supplement: Supplementary Information [file srep09733-s1.doc]

**Facile and Sustainable Synthesis of Shaped Iron Oxide Nanoparticles: Effect of Iron Precursor Salts on the Shapes of Iron Oxide**

Farheen N. Sayed, and Vivek Polshettiwar*

Nanocatalysis Laboratory (NanoCat), Department of Chemical Sciences,

Tata Institute of Fundamental Research (TIFR), Mumbai, India.

Email: [vivekpol@tifr.res.in](mailto:vivekpol@tifr.res.in)


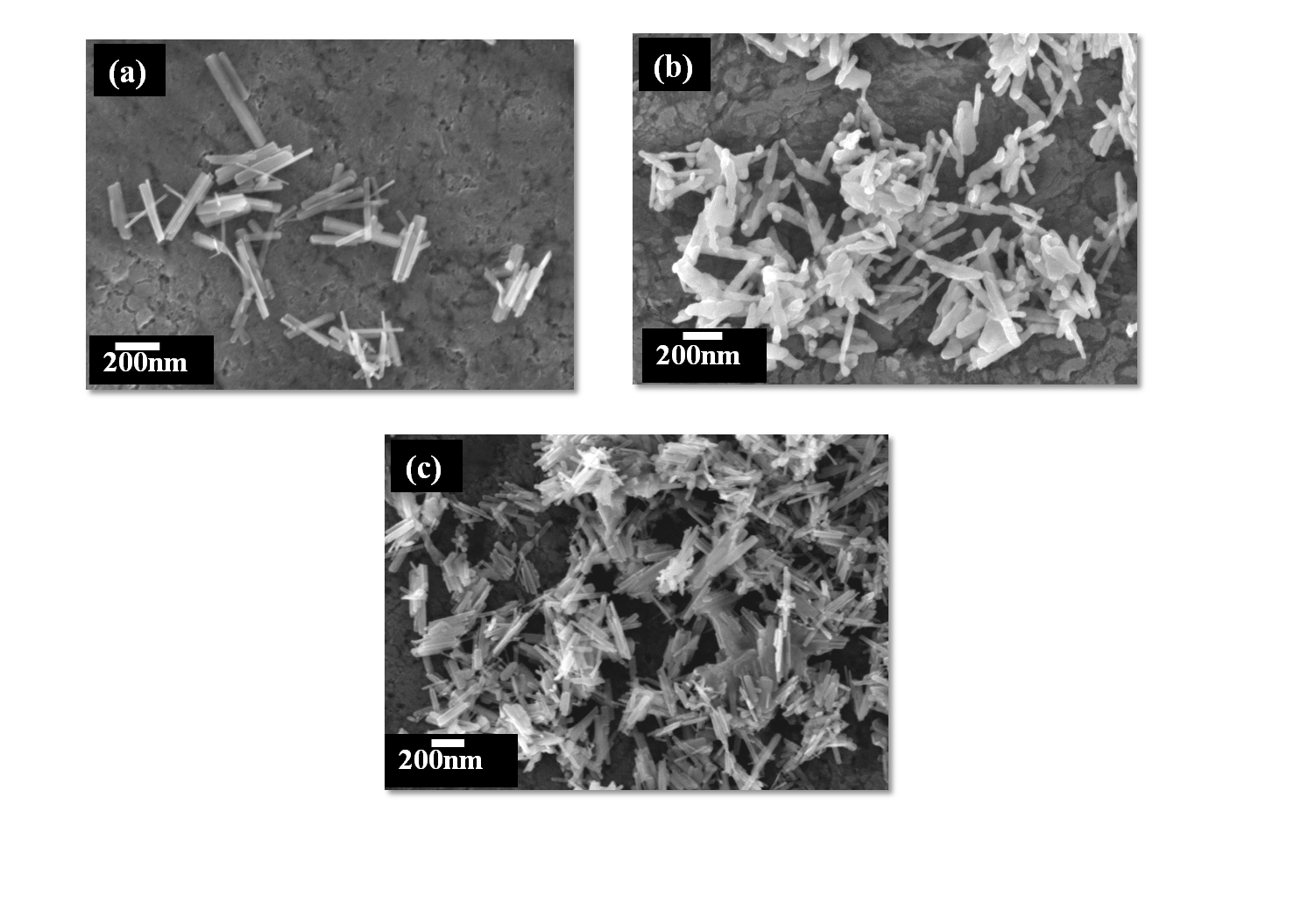


**SI-1**: SEM micrographs of heated treated nanorod samples (a)Fe-1-250-Ar, (b)Fe-1-450-Ar and (c)Fe-1-450-Air


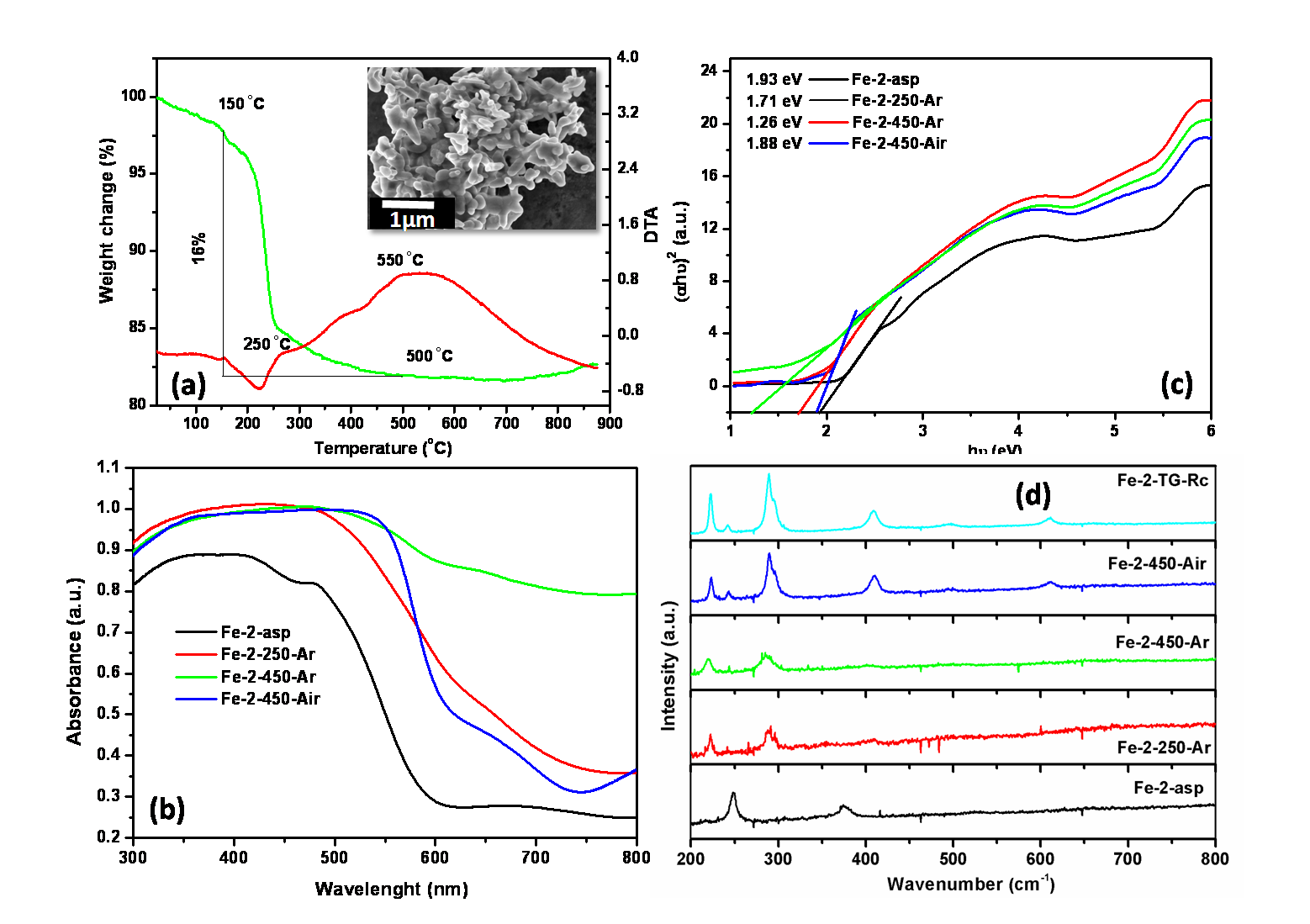


**SI- 2**: (a) TG/DTA curves of Fe-2-asp sample, inset shows the SEM image of Fe-2-TG-Rc sample; (b)absorption spectra (c)Tauc Mott plots and (d)Raman spectra of as prepared as well as heat treated samples


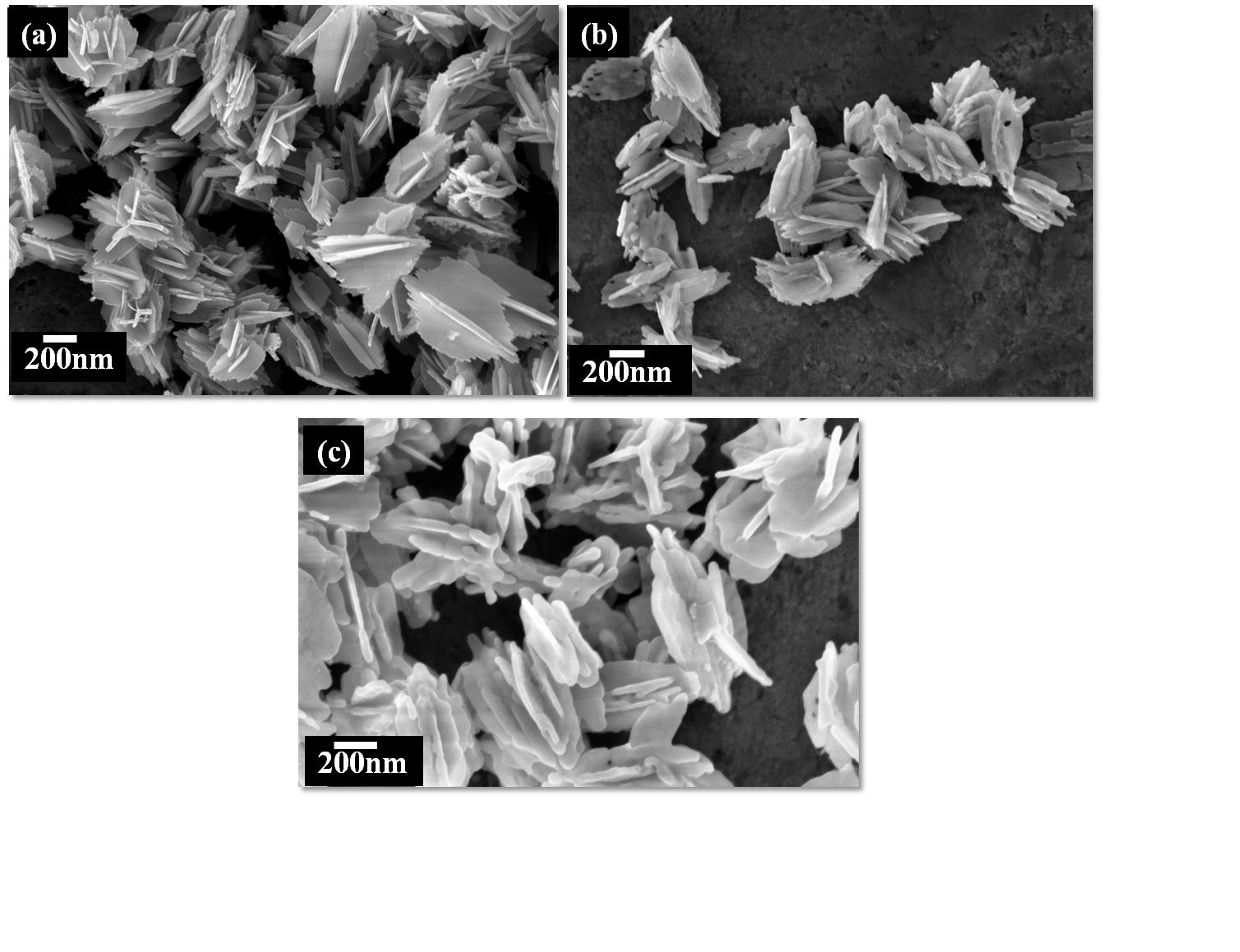


**SI-3**: SEM micrographs of heated treated nanohusk like structure (a)Fe-1-250-Ar, (b)Fe-1-450-Ar and (c)Fe-1-450-Air


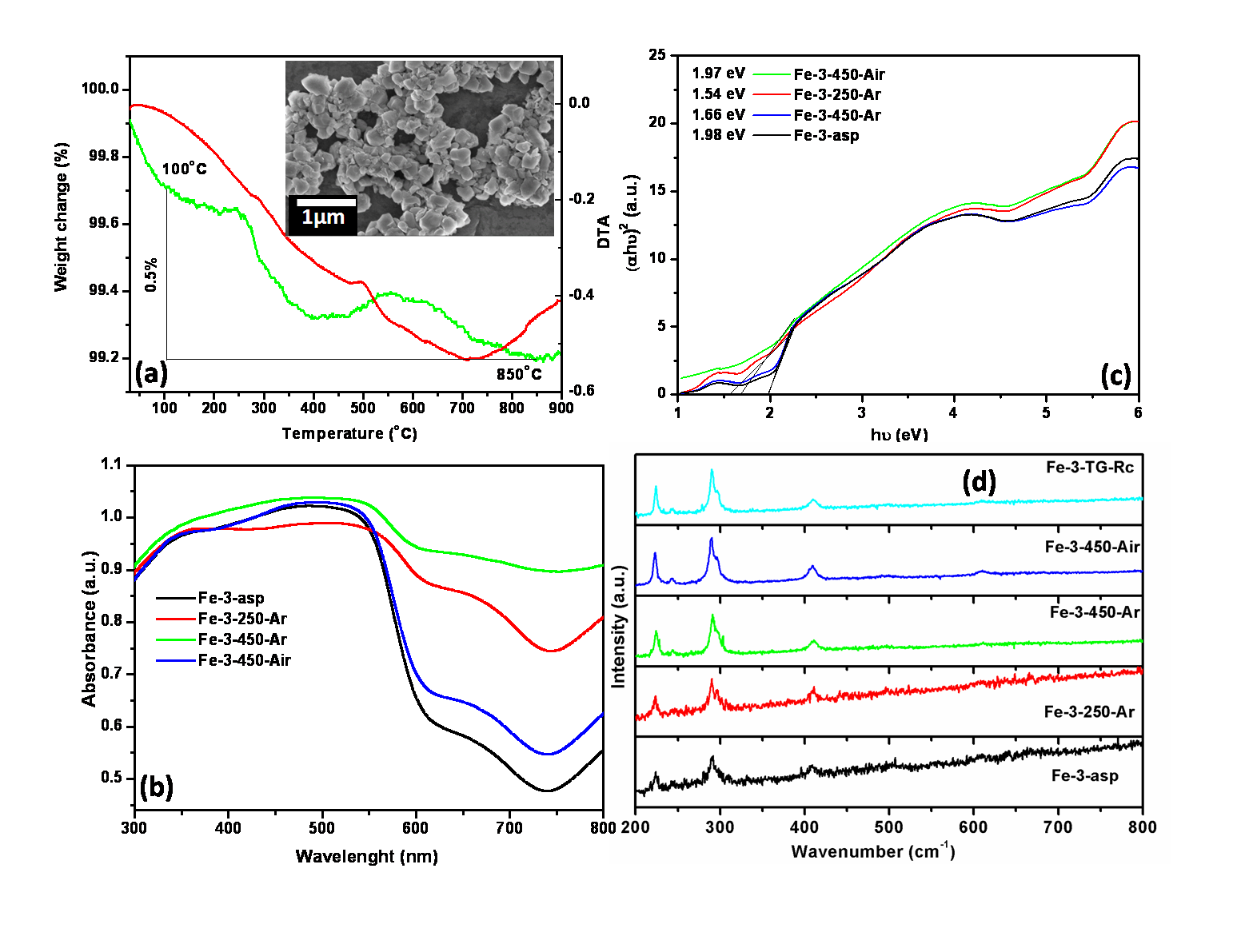


**SI- 4**: (a) TG/DTA curves of Fe-3-asp sample, inset shows the SEM image of Fe-3-TG-Rc sample; (b)absorption spectra (c)Tauc Mott plots and (d)Raman spectra of as prepared as well as heat treated samples


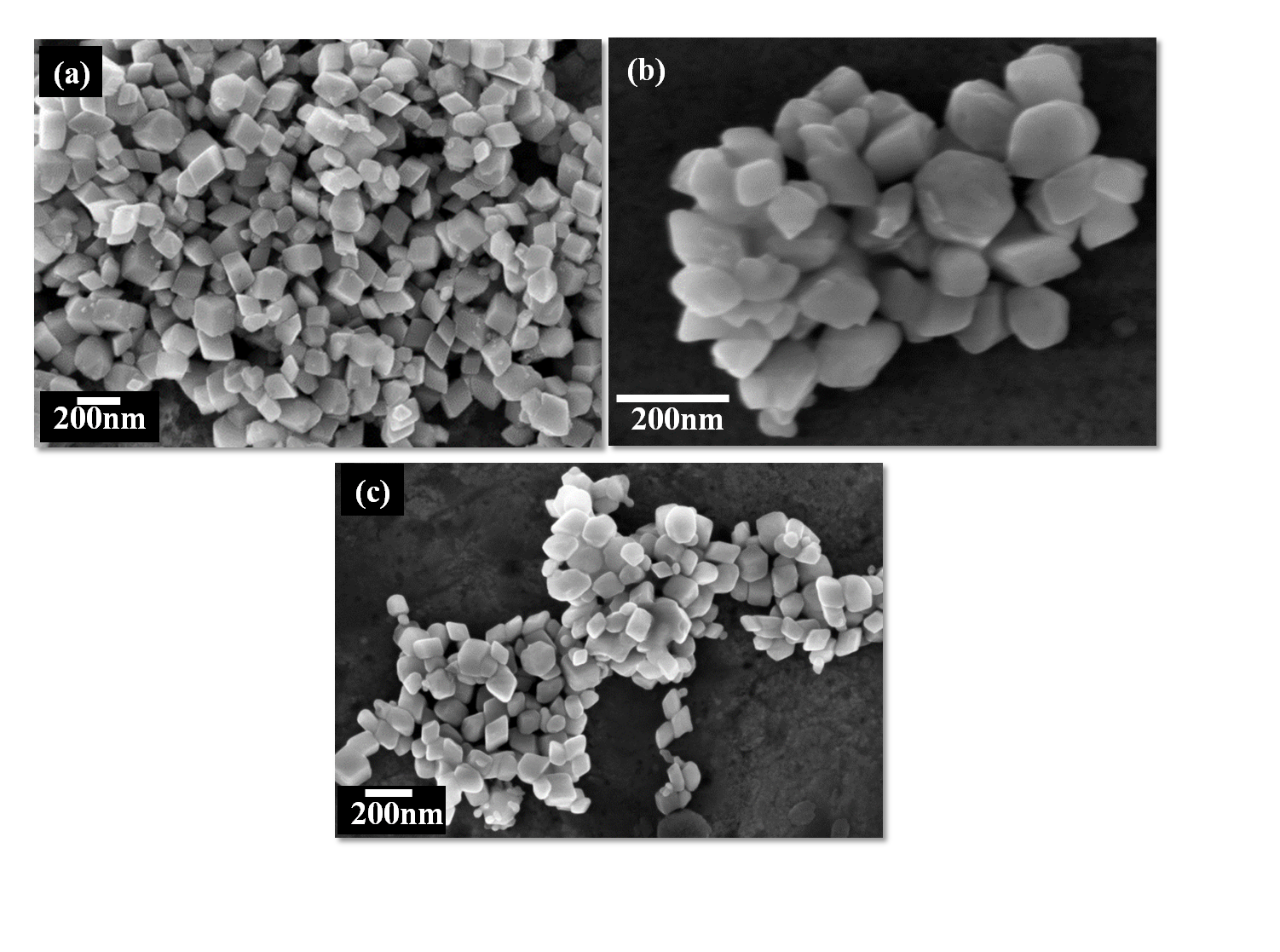


**SI-5**: SEM micrographs of heated treated distorted cubes (a)Fe-3-250-Ar, (b)Fe-3-450-Ar and (c)Fe-3-450-Air


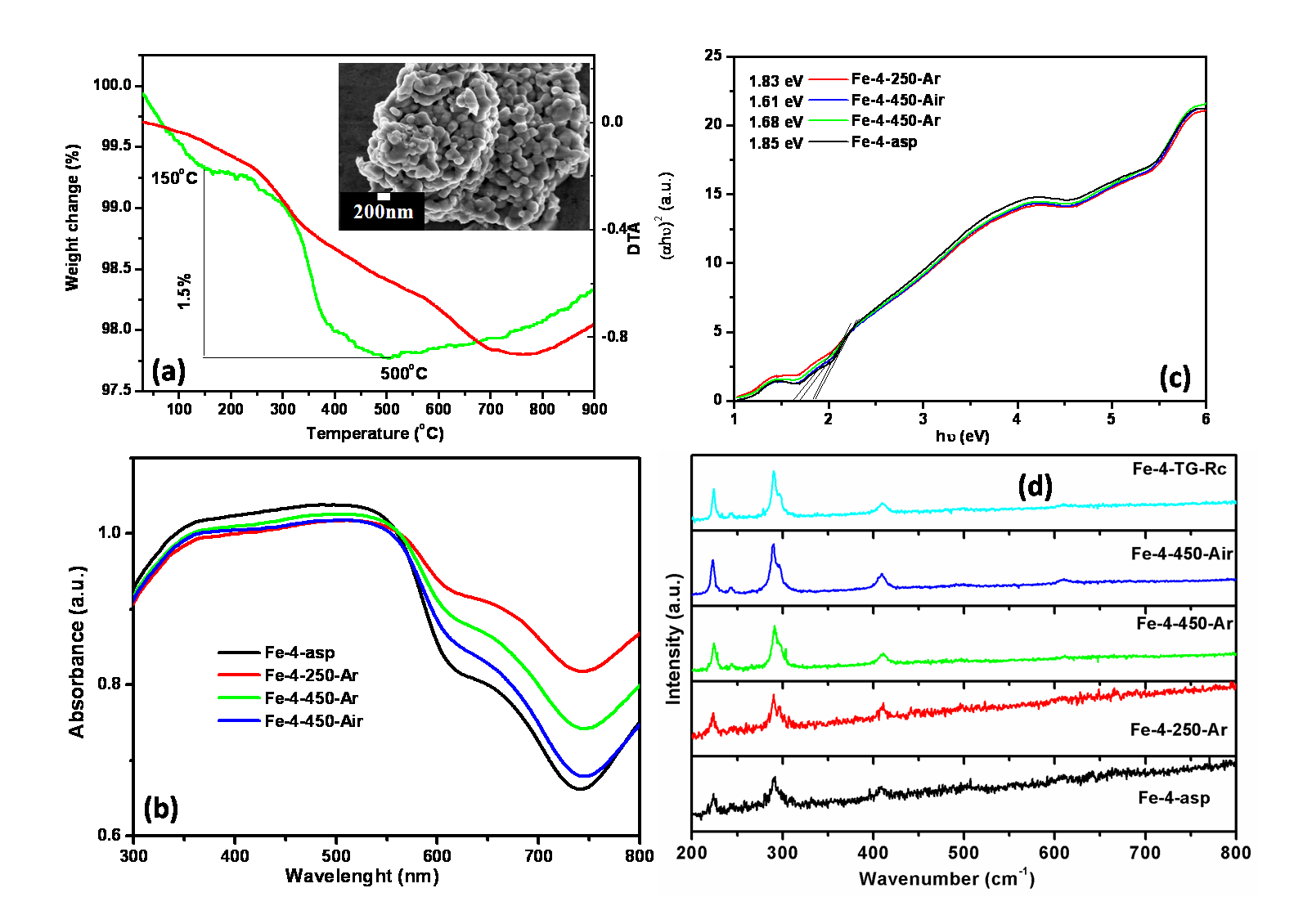


**SI- 6**: (a) TG/DTA curves of Fe-4-asp sample, inset shows the SEM image of Fe-4-TG-Rc sample; (b)absorption spectra (c)Tauc Mott plots and (d)Raman spectra of as prepared as well as heat treated samples


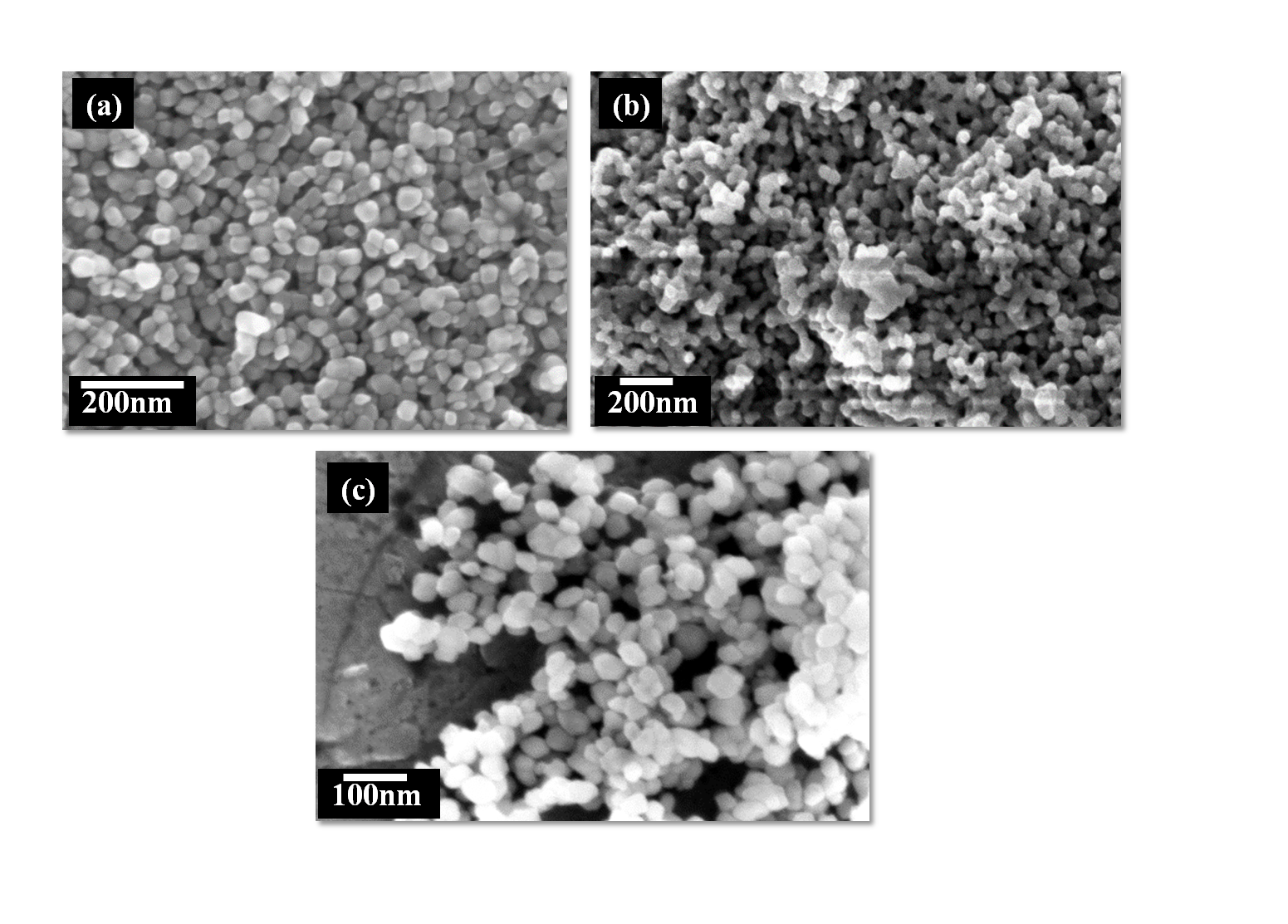


**SI-7**: SEM micrographs of heated treated nanocubes (a)Fe-4-250-Ar, (b)Fe-4-450-Ar and (c)Fe-4-450-Air


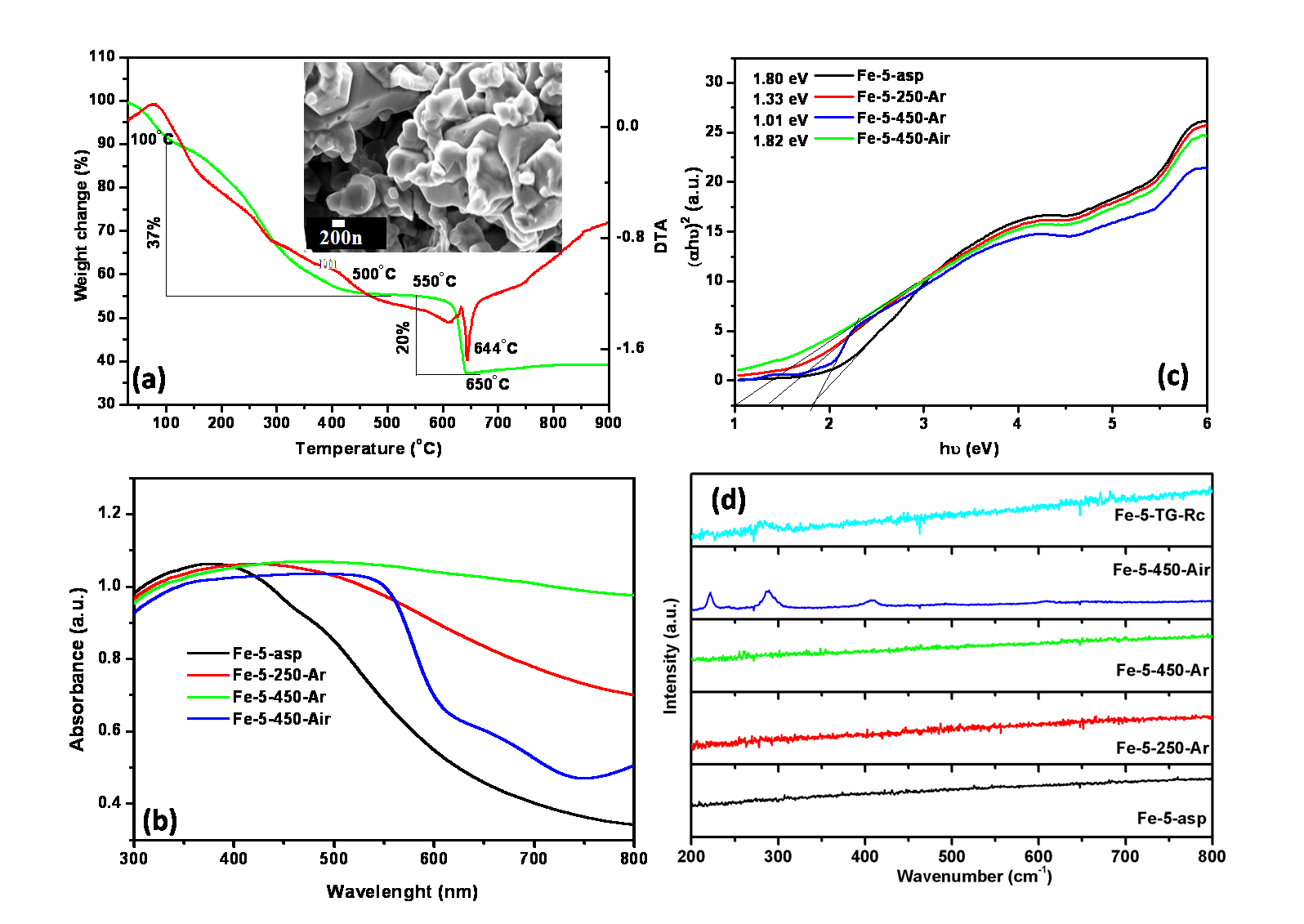


**SI- 8**: (a) TG/DTA curves of Fe-5-asp sample, inset shows the SEM image of Fe-5-TG-Rc sample; (b)absorption spectra (c)Tauc Mott plots and (d)Raman spectra of as prepared as well as heat treated samples


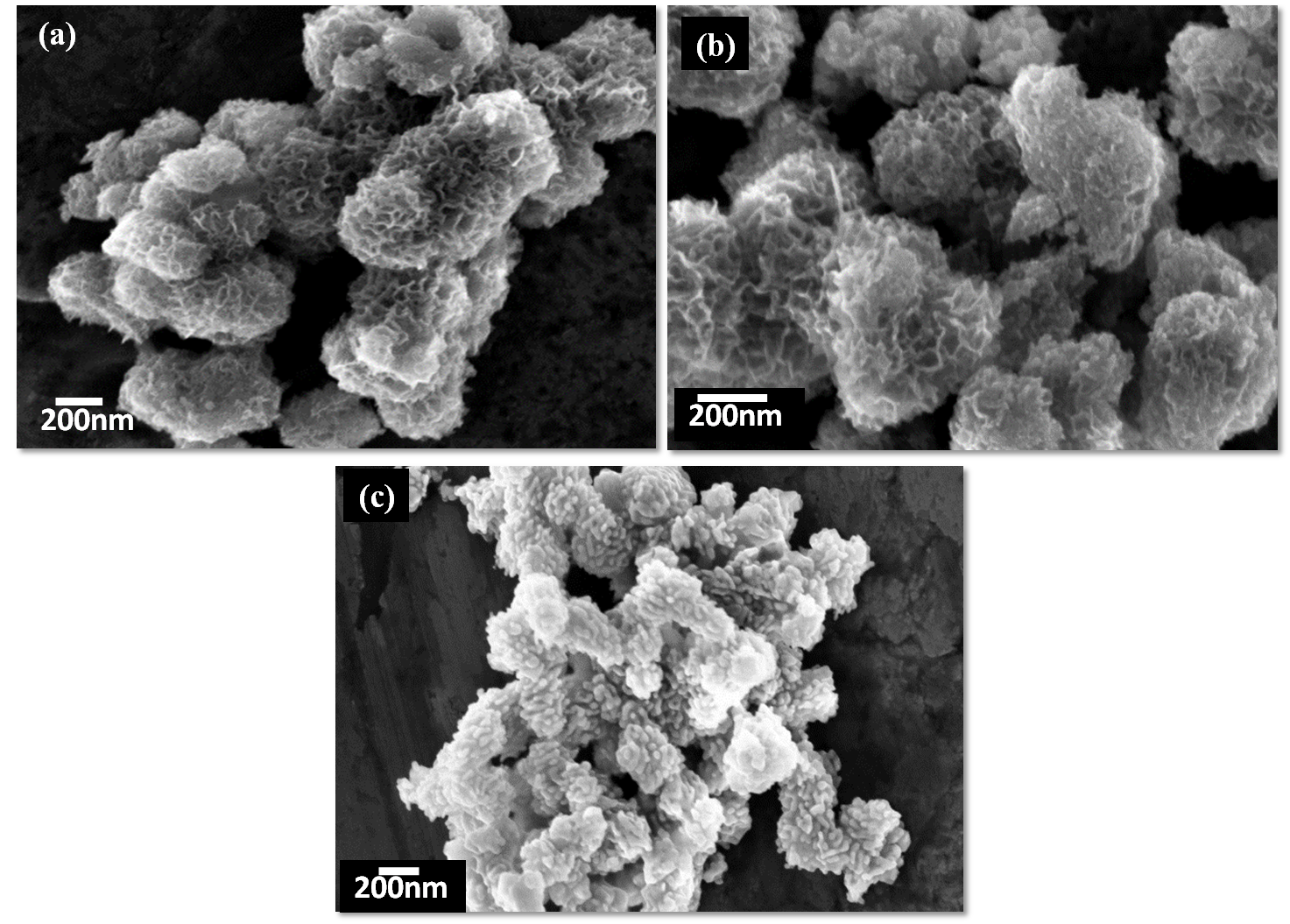


**SI-9**: SEM micrographs of heated treated porous spheres (a)Fe-5-250-Ar, (b)Fe-5-450-Ar and (c)Fe-5-450-Air


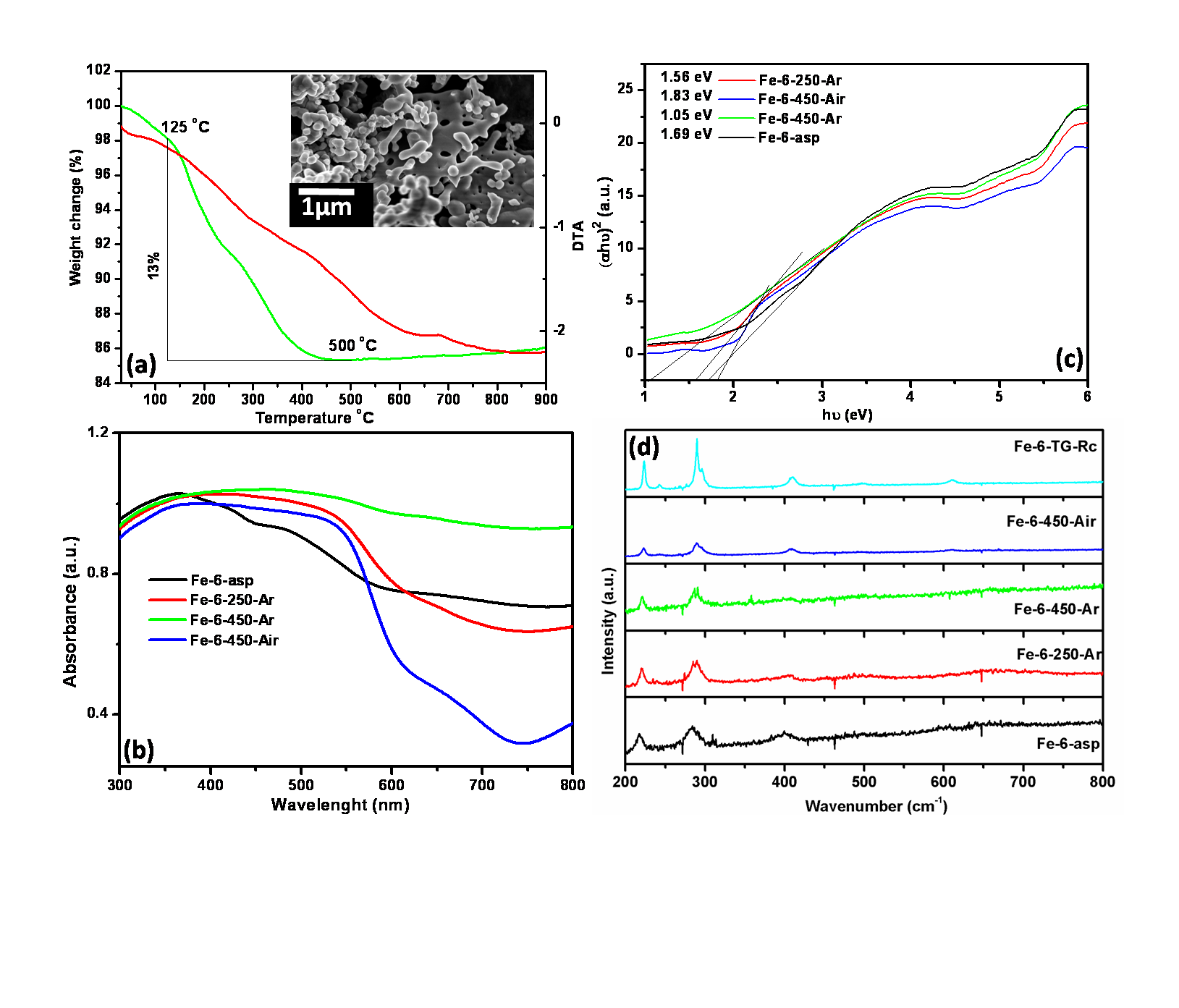


**SI- 10**: (a) TG/DTA curves of Fe-6-asp sample, inset shows the SEM image of Fe-6-TG-Rc sample; (b)absorption spectra (c)Tauc Mott plots and (d)Raman spectra of as prepared as well as heat treated samples


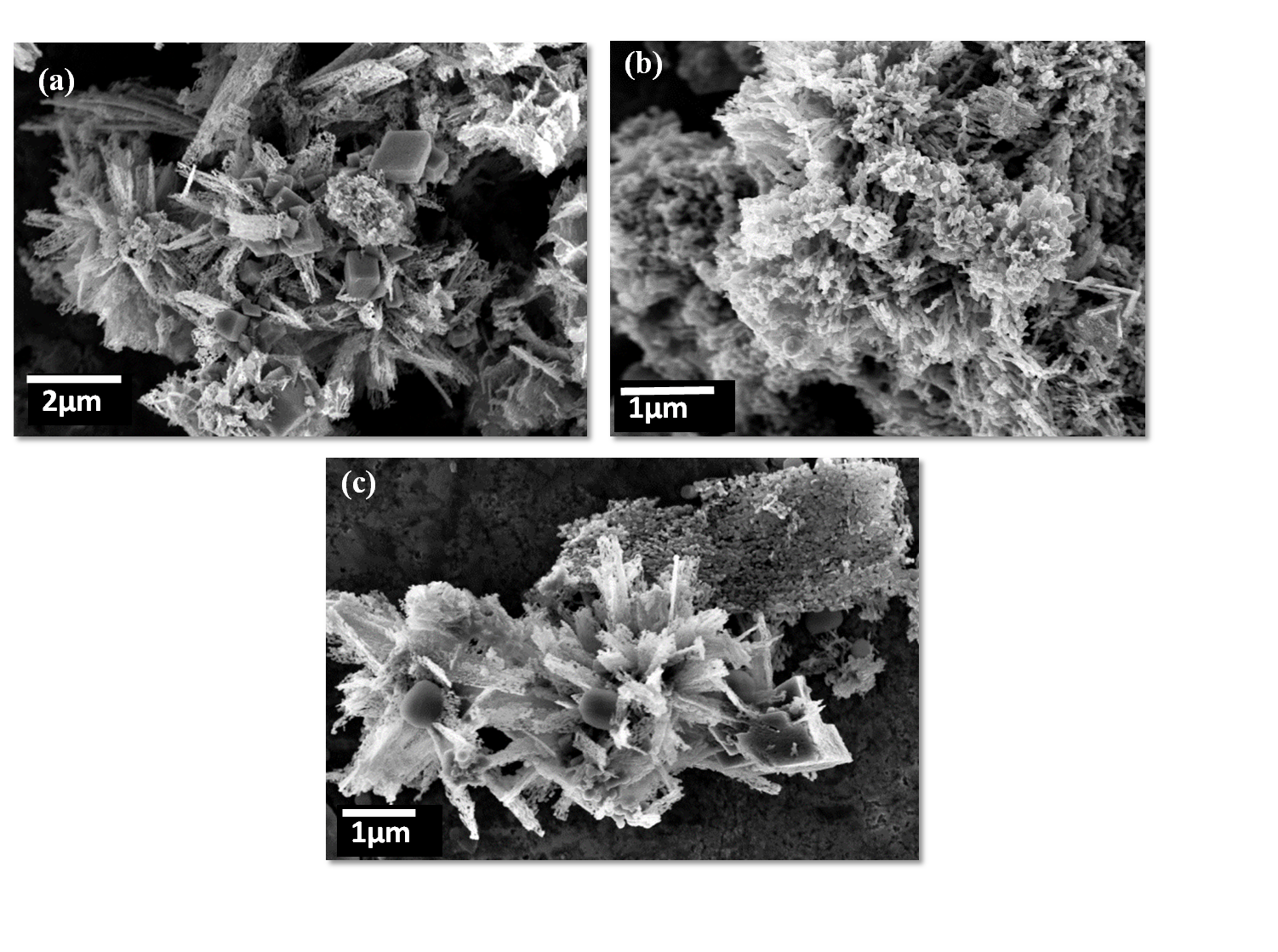
**SI-11**: SEM micrographs of heated treated self-oriented flowers (a)Fe-6-250-Ar, (b)Fe-6-450-Ar and (c)Fe-6-450-Air


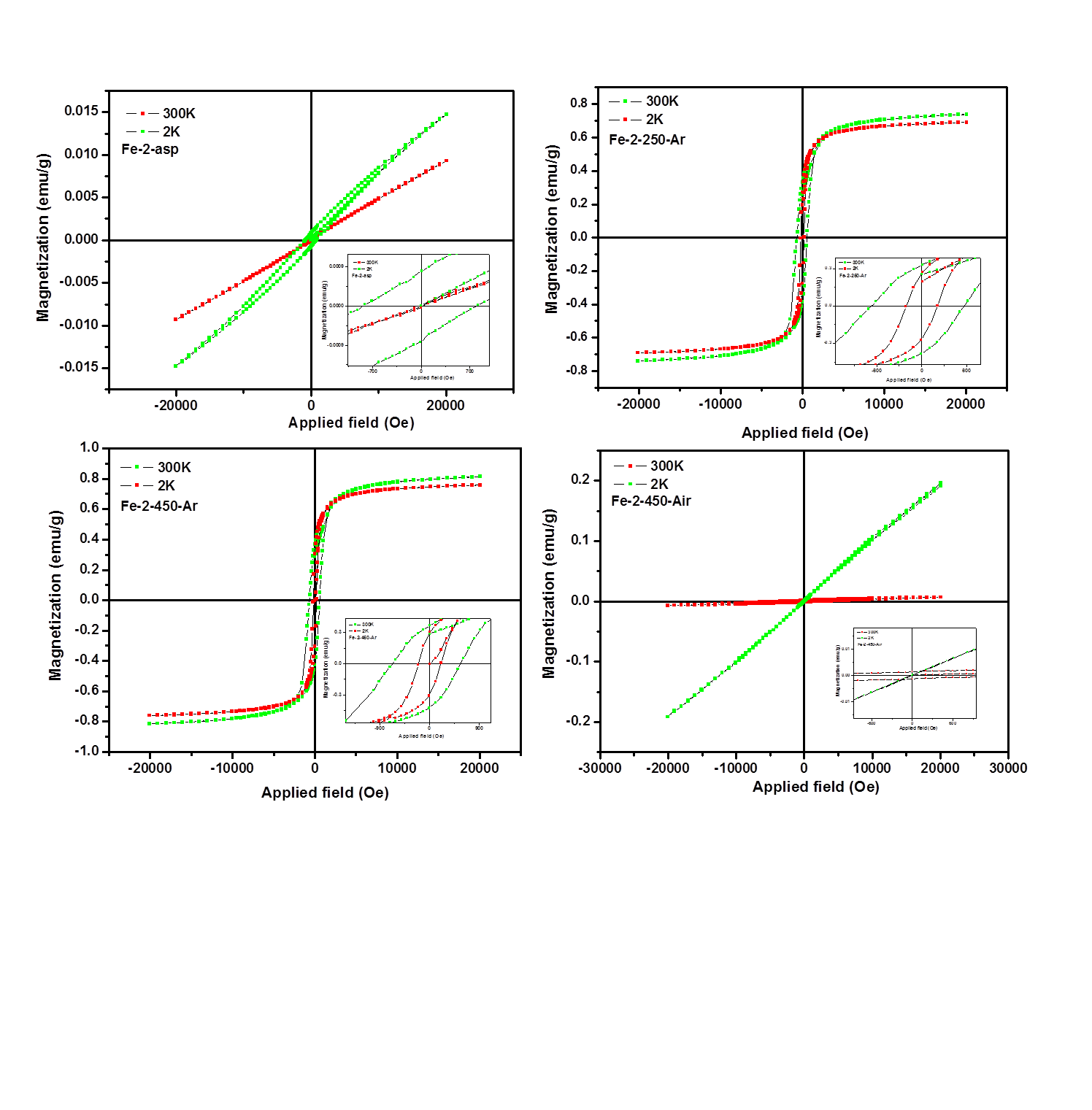


**SI-12:** Field dependent magnetization of husk like structures at 2K and 300K


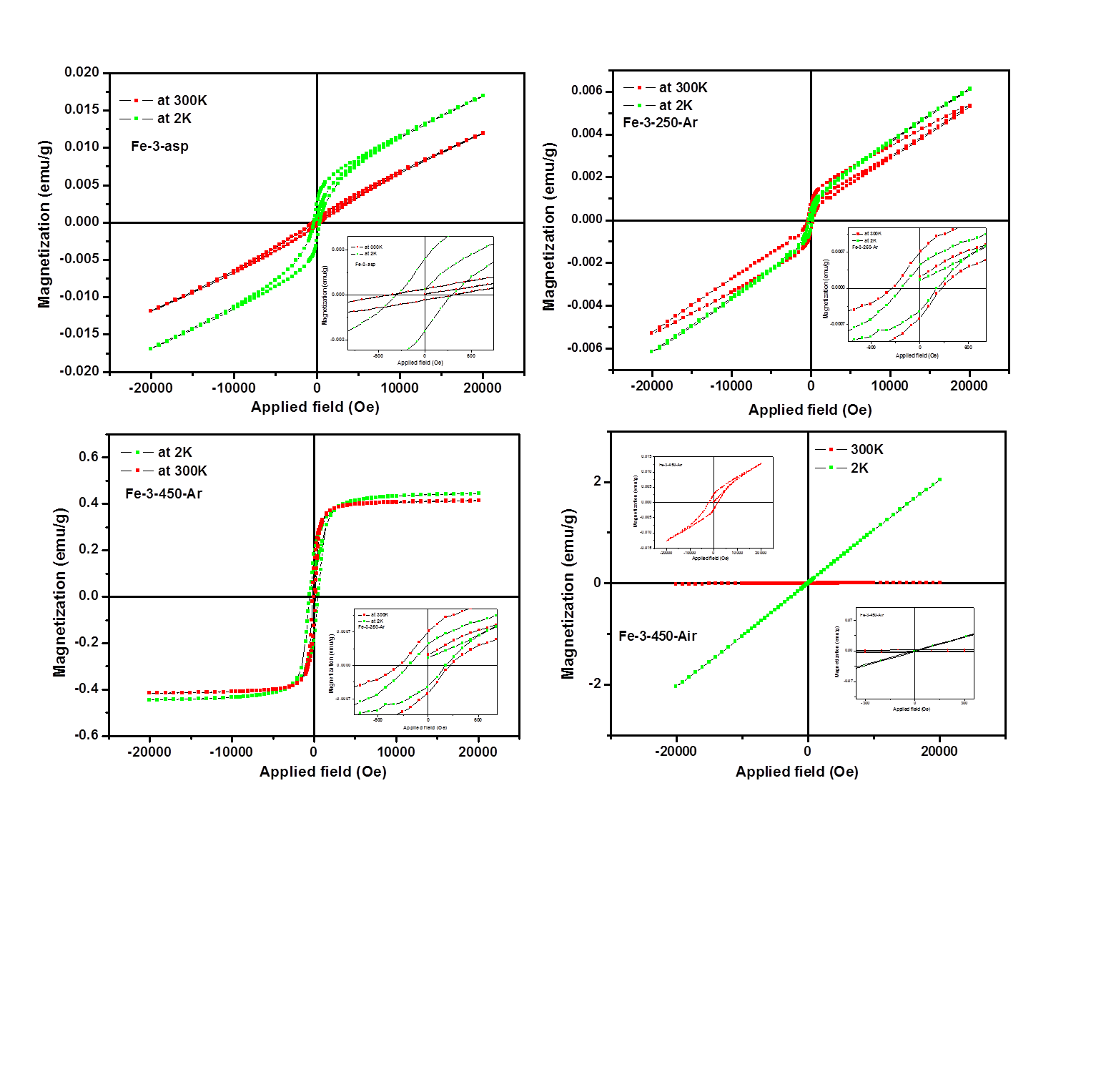


**SI-13:** Field dependent magnetization of distorted cubes at 2K and 300K

**
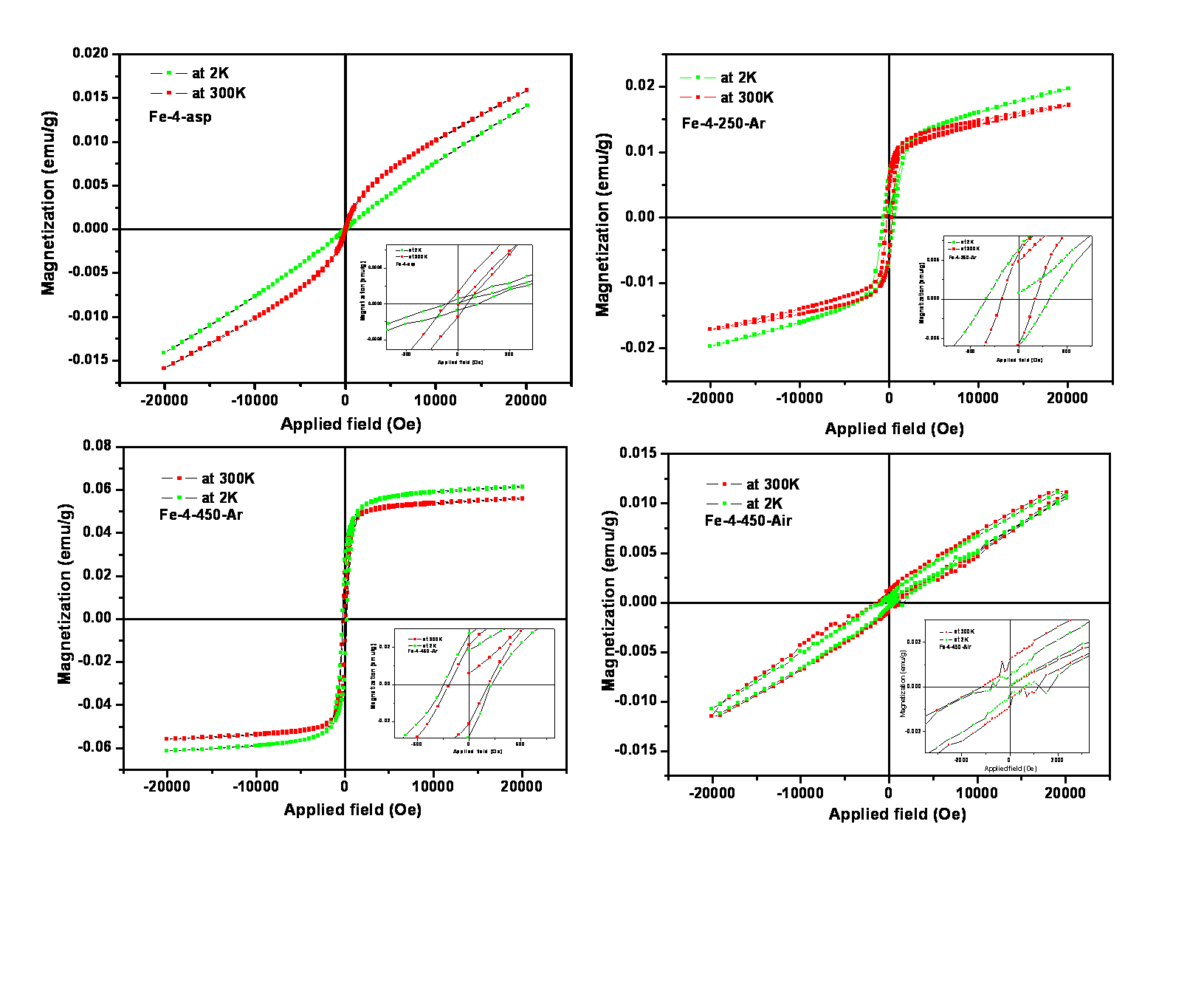
**

**SI-14:** Field dependent magnetization of nanocubes at 2K and 300K


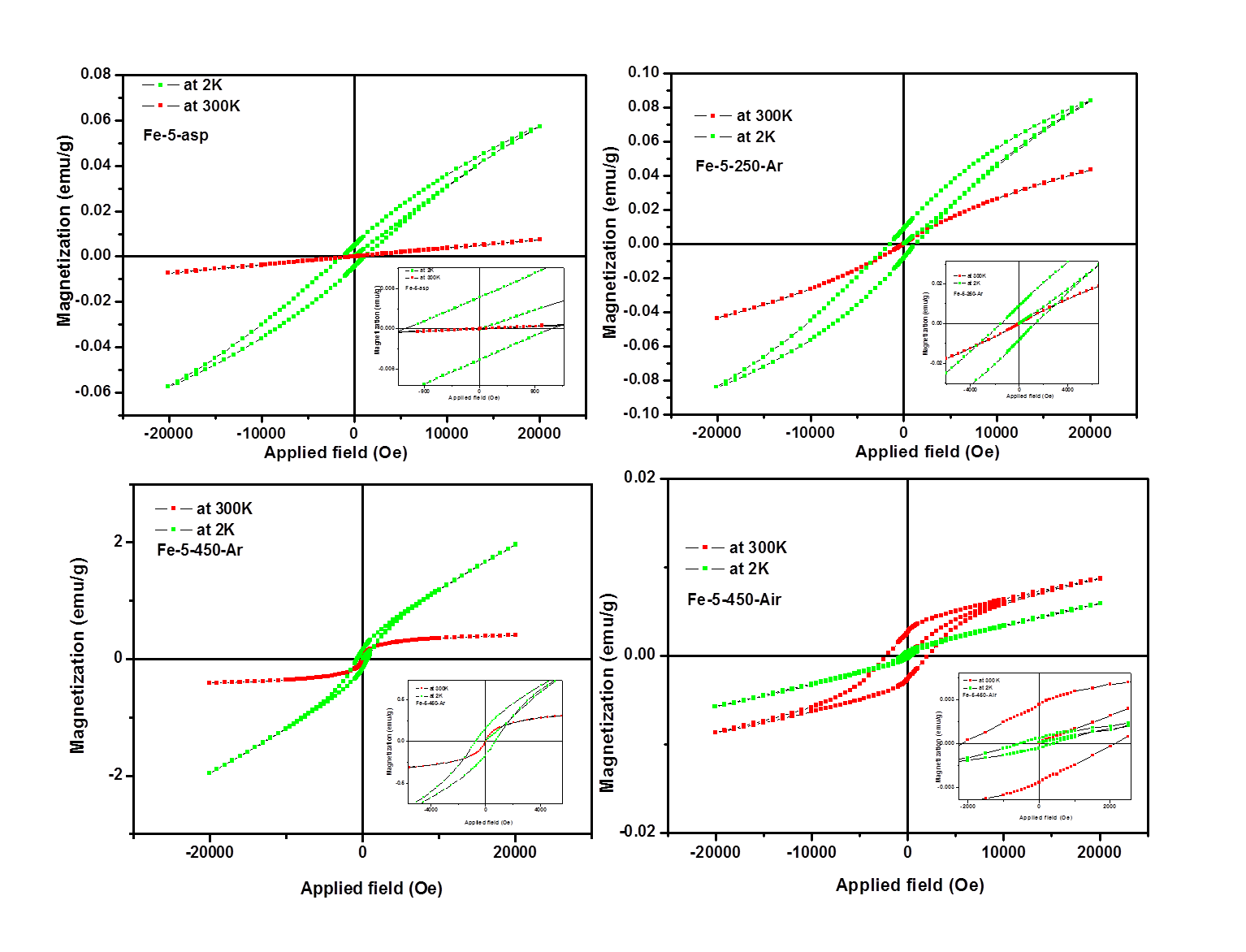


**SI-15:** Field dependent magnetization of porous spheres at 2K and 300K


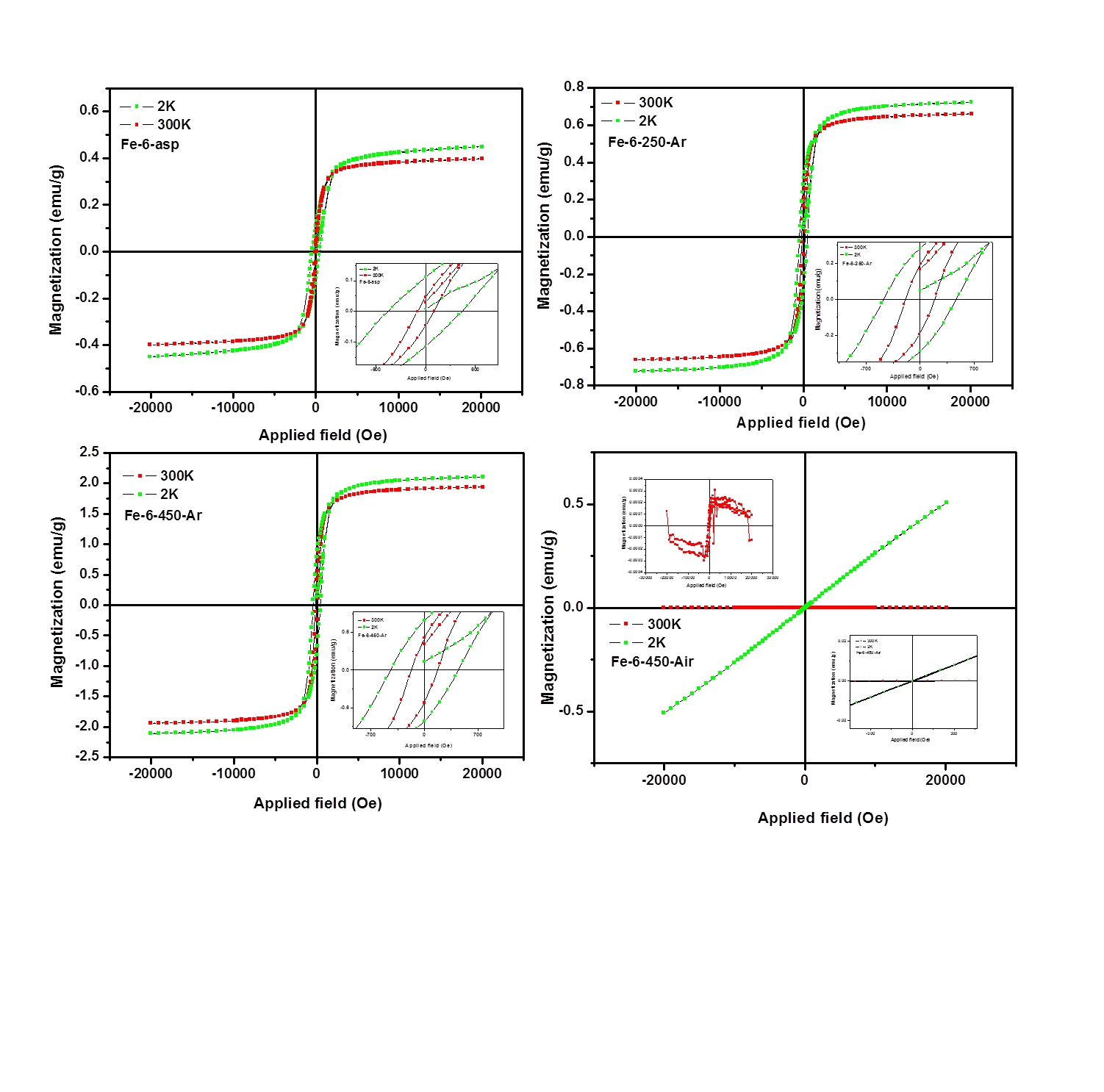


**SI-16:** Field dependent magnetization of self-oriented flowers at 2K and 300K

**SI-Table 1:** Magnetization parameters of the samples at 300K and 2K.

|  | **Saturation magnetization (M_s_), emu/g** | | **Remanant magnetization**  **(M_r_), emu/g** | | **Coercieve field**  **(H_c_), T** | |
| --- | --- | --- | --- | --- | --- | --- |
|  | **300K** | **2K** | **300K** | **2K** | **300K** | **2K** |
| **Fe-1-asp** | 0.104 | 0.113 | 0.029 | 0.045 | 231.95 | 730.91 |
| **Fe-1-250-Ar** | 0.006 | 0.009 | 4.38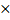10^-4^ | 8.7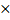10^-4^ | 82.29 | 478.68 |
| **Fe-1-450-Ar** | 0.109 | 0.118 | 0.031 | 0.053 | 236.73 | 727.29 |
| **Fe-1-450-Air** | 0.003 | 1.980 | 1.04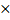10^-4^ | 8.89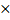10^-4^ | 123.88 | 7.468 |
| **Fe-2-asp** | 0.009 | 0.014 | 0 | 8.00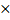10^-4^ | 0 | 784.73 |
| **Fe-2-250-Ar** | 0.689 | 0.737 | 0.261 | 0.329 | 206.18 | 563.50 |
| **Fe-2-450-Ar** | 0.762 | 0.808 | 0.300 | 0.365 | 201.03 | 561.60 |
| **Fe-2-450-Air** | 0.006 | 0.194 | 0.001 | 1.479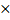10^-4^ | 2865.38 | 1.7439 |
| **Fe-3-asp** | 0.011 | 0.016 | 3.81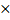10^-4^ | 2.3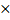10^-3^ | 413.70 | 358.20 |
| **Fe-3-250-Ar** | 0.005 | 0.006 | 7.03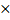10^-4^ | 4.41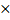10^-4^ | 263.88 | 208.10 |
| **Fe-3-450-Ar** | 0.414 | 0.444 | 0.123 | 0.18 | 169.94 | 485.08 |
| **Fe-3-450-Air** | 0.012 | 2.034 | 0.002 | 9.17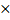10^-4^ | 1864.7 | 7.44 |
| **Fe-4-asp** | 0.015 | 0.014 | 1.966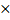10^-4^ | 8.99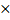10^-5^ | 51.60 | 115.89 |
| **Fe-4-250-Ar** | 0.017 | 0.019 | 5.80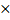10^-3^ | 6.17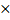10^-3^ | 311.74 | 568.23 |
| **Fe-4-450-Ar** | 0.055 | 0.061 | 2.1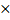10^-2^ | 2.7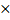10^-2^ | 187.51 | 222.21 |
| **Fe-4-450-Air** | 0.011 | 0.0108 | 11.68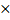10^-4^ | 4.45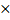10^-4^ | 1321.5 | 1835.2 |
| **Fe-5-asp** | 0.007 | 0.056 | 0 | 0.004 | 0 | 1165.12 |
| **Fe-5-250-Ar** | 0.043 | 0.084 | 0 | 0.0087 | 0 | 1307.95 |
| **Fe-5-450-Ar** | 0.008 | 0.005 | 0.002 | 3.404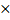10^-4^ | 2128.74 | 398.17 |
| **Fe-5-450-Air** | 0.38 | 1.96 | 0 | 0.144 | 0 | 717.51 |
| **Fe-6-asp** | 0.400 | 0.454 | 0.046 | 0.109 | 97.73 | 431.83 |
| **Fe-6-250-Ar** | 0.659 | 0.720 | 0.193 | 0.281 | 184.38 | 470.64 |
| **Fe-6-450-Ar** | 1.940 | 2.110 | 0.514 | 0.784 | 167.10 | 436.4 |
| **Fe-6-450-Air** | 7.93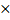10^-5^ | 0.510 | 1.32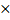10^-4^ | 1.88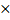10^-4^ | 273.8 | 1.93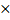10^-5^ |
